# Cardiovascular risk assessment using non-laboratory based WHO CVD risk prediction chart with respect to hypertension status among older Indian adults: insights from nationally representative survey

**DOI:** 10.3389/fpubh.2024.1407918

**Published:** 2024-09-05

**Authors:** Anshul Mamgai, Pritam Halder, Ashish Behera, Kapil Goel, Saumyarup Pal, K. S. Amudhamozhi, Divya Sharma, Tanvi Kiran

**Affiliations:** ^1^Department of Community Medicine and School of Public Health, Post Graduate Institute of Medical Education and Research (PGIMER), Chandigarh, India; ^2^Department of Internal Medicine, Post Graduate Institute of Medical Education and Research (PGIMER), Chandigarh, India; ^3^Department of Geriatric Medicine, All India Institute of Medical Sciences, New Delhi, India

**Keywords:** cardiovascular risk, non-laboratory based, WHO CVD risk prediction chart, risk prediction chart, hypertension, older Indian adults

## Abstract

**Introduction:**

Knowledge of the risk of developing cardiovascular diseases (CVD) in the population is an important risk management strategy for the prevention of this disease. This is especially true for India, which has resource-restrained settings with an increased risk in a younger population for the development of the disease. An important modifiable risk factor for CVD is hypertension, with its influence on the development of CVD.

**Methods:**

The data from the first wave of the Longitudinal Ageing Study in India (LASI) was used to calculate the 10-year CVD Risk Score among older adults ≥45 years using a WHO (2019) non-laboratory- based chart for South Asia. Univariate analysis was done using Pearson’s chi-square test, and multivariable analysis using ordinal logistic regression. Categories of CVD risk score were considered as dependent variable. Socio-demographic variables, regular exercise, history of diabetes and hyperlipidaemia were considered as the independent variables. Relationship between CVD Risk score and hypertensives and self-reported hypertensives were presented using restricted cubic splines.

**Result:**

Two-thirds (68.8%) of the population had a 10-year CVD risk of <10, and 2.8% had a risk of ≥20%. The self-reported hypertensives were distributed linearly in restricted cubic splines, with a more scattered distribution in higher scores, while actual hypertensives showed a sigmoid pattern. Urban residents (OR-0.88), being unmarried (OR-0.86), being in the richer (OR-0.94) and richest (OR-0.86) monthly *per capita* expenditure (MPCE) quintile and exercising regularly (OR-0.68) decreased the odds of being in a higher CVD risk score. Less than primary schooling (1.21) and diabetics (1.69) had higher odds for a higher CVD risk score.

**Conclusion:**

In this population, two-thirds had <10% risk for the development of CVD. The study shows a higher risk among rural, poor, and those with a lower education and lower CVD risk for those undertaking physical activity. The sigmoid pattern in actual hypertensives highlights the need for early detection. Even those with undiagnosed hypertension but with a higher BP had a similar risk for disease development, thus highlighting the need for an early detection of hypertension.

## Introduction

In 2019, of the nearly 2.5 billion healthy life years lost, 320.3 million were due to just two causes: ischemic heart disease and stroke (1). While the age-standardised deaths and Disability Adjusted Life Years (DALYs) decreased in 2019 compared to 2000, these two diseases alone added 50.2 million DALYs. The deaths due to these two diseases increased by 2.8 million from 2000 to 2019, ranking first and second respectively, throughout these years (2). Over 75% of these deaths occur in Low- and Middle- Income Countries (LMICs) (3). Simultaneously, while they face the largest burden of CVDs, they do not have sufficient resources to tackle the same (4). In India, Ischemic Heart Diseases (IHDs) and stroke account for 16.7 and 7.4% of the total deaths, respectively, and for 8 and 3.7% of the total DALYs (5). However, half the population faced catastrophic health expenditure due to hospitalization and 43% due to OPD care for cardiovascular diseases. The cost of caring for a patient with CVD in outpatient care or hospitalization is sufficient to push the population below the poverty line (6). In India, as in other LMICs, a younger working-age population is struck by cardiovascular disease, and there are more premature deaths (7, 8). Thus, it additionally leads to social and economic losses. CVD in India is estimated to cost US$ 1,044 billion by 2030 (9).

The development of cardiovascular diseases in an individual can be attributed to certain risk factors, including elevated blood pressure, tobacco consumption, obesity, poor dietary habits and a sedentary lifestyle, and increased levels of blood sugar or lipids (10). Additionally, the presence of multiple risk factors in an individual increases their risk compared to a single factor (11). Worryingly, there is a higher prevalence of CVD risk factors in the Indian population when compared to the high and upper middle-income countries (12). Prompt and appropriate interventions targeting these risk factors can mitigate the risk and also decrease the morbidity premature mortality, and disability associated with the disease and is a cost-saving strategy (10, 13). However, the identification and control of these risk factors remain limited and there is still room for improvement in adherence to cardiovascular guidelines for primary prevention (14).

The detection of levels of risk can enable the identification of a population that can benefit from treatment for CVD risk factors. A risk stratification approach is especially suitable for places with limited resources (10). The WHO has developed CVD Risk charts for 21 global regions, as delineated by the Global Burden of Disease (GBD). These charts facilitate a risk stratification approach to CVD management and are presented as laboratory-based and non-laboratory-based algorithms. This is a cost-effective strategy for preventing cardiovascular diseases in India. A two-stage screening using non-lab-based risk assessment for whole population and then screening those with ≥10% CVD risk using lab-based assessment is seen to be more cost-effective (15).

Among the modifiable risk factors for CVD, elevated blood pressure or hypertension is associated with the most compelling evidence for causation and also exhibits a high prevalence of exposure (16). Hypertension accounts for one-fifth of the CVD in the population, ranking among the highest across all income-level country groups (17). Blood pressure has a continuous, graded influence of blood pressure on the incidence and mortality of CVD (18). However, increased blood pressure rarely occurs in isolation; it is usually associated with other risk factors, further amplifying the risk due to elevated blood pressure (18). Moreover, increased blood pressure is a significant mediator for the risk of CVD due to factors such as overweight and obesity (16). Therefore, assessing the risk of CVD due to hypertension is the main theme of the present study. This study was thus done to decode the CVD risk score prescribed by the WHO and examine the influence of hypertension on this score in the Indian population using data from the Longitudinal Ageing Study in India (LASI). Further, the association of socio-demographic and disease factors with CVD Risk Score was also undertaken in the current study. The insights from this study can be utilized to develop and target interventions for the reduction of CVD. As the same population is envisioned to be followed up for the next 25 years, this study would also serve as a baseline assessment of CVD risk. This will facilitate tracking the progress over time and assess the impact of interventions and policy changes.

## Methodology

### Data source

The study utilized baseline data from the first wave of the Longitudinal Ageing Study in India (LASI). LASI surveyed older Indian adults over 45 and their spouses using a multistage stratified area probability cluster sampling design in 2017–18. Primary sampling units (PSUs) were selected based on factors like household numbers, female literacy, Scheduled Caste/Tribe population, and male non-agricultural engagement. Secondary Sampling Units (SSUs) were chosen from each PSU, proportionally allocated to rural and urban areas. A Census Enumeration Block (CEB) was randomly selected within each urban ward. Finally, households were systematically sampled from each village and CEB (19).

### Outcome variable

The outcome variable for this analysis was the CVD Risk score as defined by the World Health Organisation (WHO) in the cardiovascular disease risk non-laboratory-based chart for South Asia, published in 2019. It was calculated using the variables age, sex, smoking status, BMI category and Systolic Blood pressure category (20) (Supplementary Table S1).

The variables for the development of score were taken based on the questions asked and measurement was done as per the individual questionnaire of LASI. Age was asked as the age in completed years since their last birthday. Sex was determined by the interviewer or asked from the respondent if unclear. Those with an intake of tobacco ever were categorised as smokers. Weight was measured using digital weighing scale (Seca 803) in kilograms with 2 decimal places in light clothing. Single measurement of weight was taken as the final measurement. Height was measured using a stadiometer in centimetres standing straight without shoes and with feet together, chin tucked to chest slightly and looking straight ahead. Single measurement of height was taken. BMI was calculated using the weight and height measured during the survey. BMI was categorised as the WHO BMI Category. Blood pressure was measured using a digital BP monitor in left arm in a relaxed seating position (19). Three readings at 1 minute interval were taken and the average of the last two measures were taken as the systolic (and diastolic) blood pressure. Blood Pressure was categorised as per Joint National Commission (JNC)-8 guidelines.

A STATA .do file was developed (Supplementary material S1), to compute the WHO CVD Risk Score based on the aforementioned variables. Thus, calculated score was categorised as: < 5, 5% to <10, 10% to <20%, 20% to <30% and ≥ 30% (20, 21).

### Independent variables

The data extracted from the LASI dataset included socio-demographic characteristics such as educational status, residence, marital status, socio-economic factors including monthly *per capita* expenditure (MPCE) quintiles and health-related factors including known history of diabetes and hyperlipidemia and physical activity. Physical activity was assessed based on questions about exercise and work of the individuals, including participation in vigorous activities, moderately energetic activities, yoga, meditation, pranayama, or playing outdoor sports every day or more than once a week.

### Missing values and the final data selected

**Figure 1:**
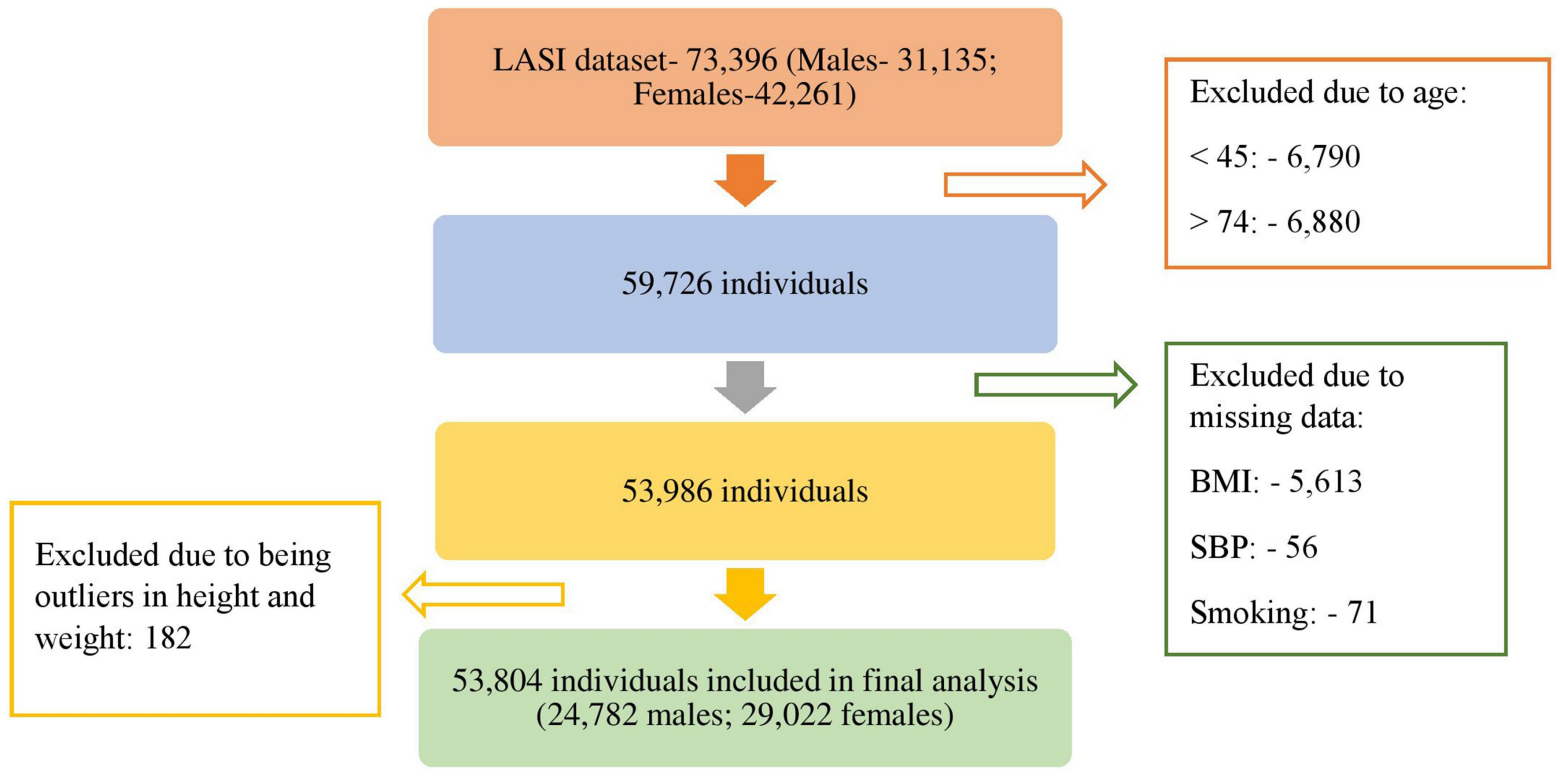
Selection of individuals for analysis.

The Longitudinal Ageing Study in India (LASI) initially included a total of 73,396 individuals, comprising 31,135 males and 42,261 females. However, individuals aged below 45 years (6,790 individuals) and above 74 years (6,880 individuals) were excluded as the analysis was focused on the age group of 45 to 74 years. Furthermore, individuals with missing information for any of the variables used in developing the cardiovascular disease (CVD) risk score were also excluded (5,740 individuals). An additional 182 individuals with outliers in weight and height were removed from the study. Consequently, a total of 53,804 individuals were included in the final analysis (Figure 1). Additionally those with a known history of stroke and heart disease were excluded from the analysis (2,480 individuals). Finally, 51,324 individuals were included in the analysis.

### Data analysis

Descriptive analysis was done using mean with standard deviation for continuous variables and frequencies with percentages. Univariate analysis was done using Pearson’s Chi-square test. The risk factors used for the development of CVD risk score were presented separately between males and females due to differences in the risk between them. For examining the association between CVD risk and socio-demographic and disease factors, we applied ordinal regression using the logit model to determine the factors associated with CVD risk based on the WHO 2019 non-laboratory-based risk score. Age, sex, smoking status, BMI category and BP were not included in the ordinal logistic based regression analysis as these were considered for developing CVD Risk score. Restricted cubic splines were used to show the graphical relationship between CVD risk score vs. self-reported hypertensive and CVD risk score vs. hypertensive. All Analysis was done using STATA 17 (StataCorp, College Station, Texas, 2021).

## Results

Supplementary Table S2 presents the socio-demographic characteristics in relation to hypertension status among males and females. It was found that 30.2% of females and 33.0% of males had high blood pressure, while 29.8% of females and 21.2% of males were known cases of hypertension. The prevalence of both high blood pressure measurements and self-reported hypertension was higher in the older age groups for both genders. The urban population exhibited a higher prevalence of self-reported hypertension and high blood pressure among both males (37.7 and 32.7% respectively) and females (28.1 and 37.1% respectively) (Supplementary Table S2). Among females, those with less than primary level schooling had the highest prevalence of high blood pressure (31.4%), while those with secondary school level of education had the highest prevalence of self-reported hypertension (36.2%). Among males, those with no schooling had the highest prevalence of high blood pressure (39.7%), while those with diploma or graduate and above had the highest prevalence of self-reported hypertension (31.5%). When classified by marital status, widowed, separated, or divorced individuals had the highest prevalence of high blood pressure and self-reported hypertension in both females (36.5 and 35.1% respectively) and males (38.8 and 23.1% respectively).

There was no significant difference in the prevalence of high blood pressure among females in the different MPCE quintiles, but the prevalence of self-reported hypertension increased from 23.3% in the poorest quintile to 35.1% in the richest quintile. Similar trends were observed for males. Both females and males who exercised regularly had a lower prevalence of high blood pressure (29.9 and 32.1%) and self-reported hypertension (28.8 and 20.0%) when compared to those who did not exercise regularly. Those with a history of smoking had a higher prevalence of high blood pressure in females (31.0%); the non-smokers had a higher prevalence in males (35.3%). The non-smokers also had a higher prevalence of self-reported hypertension in both females (30.5%) and males (25.6%). Self-reported hypertension was most prevalent among morbidly obese females (54.9%) and obese males (40.1%). A higher prevalence of high blood pressure was observed among obese individuals in both females (38.3%) and males (47.9%). Those with known diabetes had a higher prevalence of high blood pressure and self-reported hypertension among both females (38.9 and 62.4%) and males (40.9 and 53.0%), compared to those without any such history. A similar situation was observed with hypercholesterolemia.

Supplementary Table S3 shows the distribution of the study population with respect to the risk factors used for the development of the cardiovascular disease (CVD) risk score. Between one-fifth and one-fourth of the population, both females (23.7%) and males (22.4%), were 45–49 years old. Most females (80.5%) were non-smokers, while over half of the males (56.4%) were smokers. Most of the population, including both male and female subgroups, had a normal Body Mass Index (BMI). Two-fifths of the population had a systolic blood pressure of 120–139 mmHg.

Two-third (68.7%) of the population had a 10-year fatal and non-fatal cardiovascular disease (myocardial infarction and stroke) risk of <10% (with 29.8% having a risk of <5%), 28.5% of the participants had a risk of 10- < 20 and 2.7% had a risk of ≥20%. A CVD risk score of 30% or higher was observed in individuals aged above 65 years, smokers, and those with systolic blood pressure above 160 mmHg. This was distributed across both genders and all BMI categories. Compared to the rural population, the urban population had a slightly higher proportion in the <5% group, and slightly lower proportion in 5- < 10%, 10- < 20%, and 20- < 30% groups. Among education groups, individuals with less than primary level schooling had the lowest proportion in the less than 5% risk group (25.8%). Those educated up to higher secondary level had the highest proportion in the less than 5% risk group (38.6%) and the diploma holders of graduates and above had the highest proportion in the 5- < 10% risk group (36.1%).

Unmarried individuals had a higher proportion in the less than 5% risk group (37.2%) compared to married (32.5%) and widowed/separated/divorced individuals (19.3%). The population belonging to the richest MPCE quintile had the highest proportion in the less than 5% CVD risk score group (32.5%) in the population when divided by the MPCE quintile.

Those who exercised regularly had a higher proportion in the lower CVD risk score groups of <5% (31.8%) and 5- < 10% (39.5%) compared to those who did not exercise regularly. Diabetics and those with high cholesterol had a lower prevalence in these groups compared to those without these conditions (Table 1).

**Table 1:** Distribution of participants as per CVD risk score.

Total	Total population	<5%, *N* (%)	5– < 10%, *N* (%)	10– < 20%, *N* (%)	20– < 30%, *N* (%)	≥30%, *N* (%)
*Age group*						
45–49	11,861 (23.1)	9,165 (77.3)	2,580 (21.8)	116 (1.0)	0	0
50–54	9,686 (18.9)	4,655 (48.1)	4,471 (46.2)	559 (5.8)	1 (0.0)	0
55–59	8,778 (17.1)	1,544 (17.6)	5,680 (64.7)	1,518 (17.3)	36 (0.4)	0
60–64	8,785 (17.1)	0	5,338 (60.8)	3,392 (38.6)	55 (0.6)	0
65–69	7,476 (14.6)	0	1,874 (25.1)	5,220 (69.8)	380 (5.1)	2 (0.0)
70–74	4,738 (9.2)	0	0	3,817 (80.6)	889 (18.8)	32 (0.7)
*Gender*						
Female	27,903 (53.9)	10,715 (38.4)	10,927 (39.2)	5,889 (21.1)	372 (1.3)	3 (0.0)
Male	23,418 (46.1)	4,649 (19.9)	9,016 (38.5)	8,733 (37.3)	989 (4.2)	31 (0.1)
*Smoking status*						
Non-Smoker	32,667 (63.7)	13,763 (42.1)	12,436 (38.1)	6,293 (19.3)	175 (0.5)	0
Smoker	18,657 (36.3)	1,601 (8.6)	7,507 (40.2)	8,329 (44.6)	1,186 (6.4)	34 (0.2)
*BMI category*						
< 20	14,906 (29.0)	3,957 (26.6)	6,125 (41.1)	4,413 (29.6)	408 (2.7)	3 (0.0)
20–24	21,025 (41.0)	6,591 (31.4)	7,820 (37.2)	6,132 (29.2)	474 (2.3)	8 (0.0)
25–29	11,606 (22.6)	3,442 (29.7)	4,653 (40.1)	3,105 (26.8)	387 (3.3)	19 (0.2)
30–35	3,137 (6.1)	1,156 (36.9)	1,103 (35.2)	797 (25.4)	77 (2.5)	4 (0.1)
≥ 35	650 (1.3)	218 (33.5)	242 (37.2)	175 (26.9)	15 (2.3)	0
*Systolic blood pressure*						
< 120	18,725 (36.5)	9,348 (49.9)	6,810 (36.4)	2,567 (13.7)	0	0
120–139	20,112 (39.2)	5,285 (26.3)	8,678 (43.2)	6,073 (30.2)	76 (0.4)	0
140–159	9,139 (17.8)	731 (8.0)	3,630 (39.7)	4,176 (45.7)	602 (6.6)	0
160–179	3,160 (6.2)	0	803 (25.4)	1,718 (54.4)	620 (19.6)	19 (0.6)
≥ 180	188 (0.4)	0	22 (11.7)	88 (46.8)	63 (33.5)	15 (8.0)
*Residence*						
Urban	17,373 (33.9)	5,474 (31.5)	6,657 (38.3)	4,829 (27.8)	403 (2.3)	10 (0.1)
Rural	33,951 (66.1)	9,890 (29.1)	13,286 (39.1)	9,793 (28.8)	958 (2.8)	24 (0.1)
*Education*						
No schooling	23,681 (46.1)	6,573 (27.8)	9,437 (39.9)	7,006 (29.6)	650 (2.7)	15 (0.1)
Less than primary (till 4)	5,841 (11.4)	1,505 (25.8)	2,240 (38.4)	1,904 (32.6)	186 (3.2)	6 (0.1)
Primary completed (5–7)	6,959 (13.6)	2,112 (30.4)	2,700 (38.8)	1,950 (28.0)	193 (2.8)	4 (0.1)
Middle completed (8–9)	5,136 (10.0)	1,741 (33.9)	2,003 (39.0)	1,277 (24.9)	110 (2.1)	5 (0.1)
Secondary school (10–11)	4,658 (9.1)	1,530 (32.9)	1,748 (37.5)	1,248 (26.8)	128 (2.8)	4 (0.1)
Higher secondary	2,255 (4.4)	870 (38.6)	807 (35.8)	530 (23.5)	48 (2.1)	0
Diploma or graduate and above	2,794 (5.4)	1,033 (37.0)	1,008 (36.1)	707 (25.3)	46 (1.7)	0
*Marital status*						
Unmarried	651 (1.3)	242 (37.2)	250 (38.4)	148 (22.7)	11 (1.7)	0
Married/ in live -in	40,420 (78.)	13,146 (32.5)	15,905 (39.4)	10,438 (25.8)	907 (2.2)	24 (0.1)
Widow/ separated/ divorced	10,252 (20.0)	1,976 (19.3)	3,787 (36.9)	4,036 (39.4)	443 (4.3)	10 (0.1)
*MPCE quintile*						
Poorest	10,286 (20.0)	2,904 (28.2)	4,029 (39.2)	3,048 (29.6)	301 (2.9)	4 (0.0)
Poorer	10,460 (20.4)	2,956 (28.3)	4,170 (39.9)	3,038 (29.0)	292 (2.8)	4 (0.0)
Middle	10,372 (20.2)	3,136 (30.2)	3,930 (37.9)	3,027 (29.2)	272 (2.6)	7 (0.1)
Richer	10,264 (20.0)	3,135 (30.5)	3,963 (38.6)	2,879 (28.1)	274 (2.6)	13 (0.1)
Richest	9,942 (19.4)	3,233 (32.5)	3,851 (38.7)	2,630 (26.5)	222 (2.2)	6 (0.1)
*Regular exercise*						
No	13,298 (25.9)	3,264 (24.6)	4,933 (37.1)	4,600 (34.6)	484 (3.6)	17 (0.1)
Yes	38,026 (74.1)	12,100 (31.8)	15,010 (39.5)	10,022 (26.4)	877 (2.3)	17 (0.0)
*Known diabetes*						
No	45,262 (88.2)	14,092 (31.1)	17,567 (38.8)	12,427 (27.5)	1,147 (2.5)	29 (0.1)
Yes	6,046 (11.8)	1,267 (21.0)	2,369 (39.2)	2,191 (36.2)	214 (3.5)	5 (0.1)
*Known hypercholesterolemia*						
No	49,804 (97.1)	14,933 (30.0)	19,326 (38.8)	14,193 (28.5)	1,319 (2.7)	33 (0.1)
Yes	1,514 (2.9)	430 (28.4)	613 (40.5)	428 (28.3)	42 (2.8)	1 (0.1)
Total	51,324	15,364 (29.8)	19,943 (38.9)	14,622 (28.5)	1,361 (2.7)	34 (0.1)

When categorised by sex, it was observed that most women aged between 45 and 49 years and 50–54 years had a risk of less than 5% (88.3%). Among men aged between 45 to 49 years, the majority (63.4%) had a risk of less than 5%, while one-fourth of men between 50–54 years had a risk of less than 5% (26.5%). Majority of females (65.6%) and males (63.6%) aged between 55 and 59 years had a risk of 5- <10. In the age group of 60–64 years, three-fourths of females had a risk between 5- < 10% (77.8%), while most males had a risk between 10- < 20% (58.8%). Half of females aged between 65 and 69 years (57.1%) and most males (83.4%) had a risk between 10% to less than 20%. Among those aged between 70 and 74 years, most females (89.2%) had a risk between 10- < 20%, while one-fourth of males (27.0%) had a risk between 20- < 30% (Supplementary Table S4).

Tables 2, 3 display the CVD risk among individuals with and without controlled blood pressure during the survey, categorised by their known hypertension status and age group. In the population with blood pressure below the hypertension level, a higher proportion (40.1%) was observed in the lowest risk group of less than 5% among those without a known history of hypertension, compared to those with a known history of hypertension (30.9%). However, in the age groups 45–49 and 50–54, a higher prevalence was seen in those with known hypertension. Among those with blood pressure above the hypertension range, a higher proportion in the lowest risk group of less than 5% was seen among those without a known history of hypertension (13.5%), compared to those with a known hypertension status (9.4%). In the age groups 45–49 years, those with a known hypertension; and in 50–54 years, those without any known history of hypertension had a higher proportion in the <5% Risk score category. Gender-wise classification of CVD Risk Score among participants with uncontrolled blood pressure was documented in Supplementary Tables S5, S6.

**Table 2:** CVD risk among the participants with blood pressure below the diagnostic level of hypertension.

Age-group (years)	Self-reported hypertension present						Self-reported hypertension absent					
	Total	<5% (%)	5–9% (%)	10–19% (%)	20–29% (%)	≥30% (%)	Total	<5% (%)	5–9% (%)	10–19% (%)	20–29% (%)	≥30% (%)
45–49	1,287 (17.2)	1,114 (86.6)	173 (13.4)	0	0	0	7,651 (27.6)	6,543 (85.5)	1,103 (14.4)	5 (0.1)	0	0
50–54	1,288 (17.2)	897 (69.6)	387 (30.0)	4 (0.3)	0	0	5,611 (20.3)	3,316 (59.1)	2,280 (400.6)	15 (0.3)	0	0
55–59	1,301 (17.4)	296 (22.8)	888 (68.3)	117 (9.0)	0	0	4,707 (17.0)	1,235 (26.6)	3,015 (64.1)	457 (9.7)	0	0
60–64	1,404 (18.8)	0	1,070 (76.2)	334 (23.8)	0	0	4,328 (15.6)	0	3,111 (71.9)	2,039 (60.2)	0	0
65–69	1,330 (17.8)	0	474 (350.6)	854 (64.2)	2 (0.2)	0	3,385 (12.2)	0	1,346 (39.8)	2,039 (66.2)	0	0
70–74	869 (11.6)	0	0	840 (96.7)	29 (3.3)	0	2,018 (7.3)	0	0	1,979 (98.1)	39 (1.9)	0
All groups	7,479 (21.2)	2,307 (30.9)	2,992 (40.0)	2,149 (28.7)	31 (0.4)	0	27,700 (78.8)	11,094 (40.1)	10,855 (39.2)	5,712 (20.6)	39 (0.1)	0

**Table 3:** CVD risk among the participants with blood pressure above the diagnostic level of hypertension.

Age-group (years)	Self-reported hypertension present						Self-reported Hypertension absent					
	Total	<5% (%)	5–9% (%)	10–19% (%)	20–29% (%)	≥30% (%)	Total	<5% (%)	5–9% (%)	10–19% (%)	20–29% (%)	≥30% (%)
45–49	866 (14.9)	449 (51.9)	369 (42.6)	48 (5.5)	0	0	2,052 (19.9)	1,056 (51.5)	933 (45.5)	63 (3.1)	0	0
50–54	916 (15.7)	119 (13.0)	608 (66.4)	188 (20.5)	1 (0.1)	0	1,870 (18.1)	323 (17.3)	1,195 (63.9)	352 (18.8)	0	0
55–59	991 (17.0)	3 (0.3)	658 (66.4)	308 (31.1)	22 (2.2)	0	1,777 (17.2)	9 (0.5)	1,119 (63.0)	635 (35.7)	14 (0.8)	0
60–64	1,119 (19.2)	0	431 (38.5)	657 (58.7)	31 (2.8)	0	1,930 (18.7)	0	723 (37.5)	1,813 (61.3)	24 (1.2)	0
65–69	1,161 (20.0)	0	16 (1.4)	976 (58.7)	168 (14.5)	1 (0.1)	1,599 (15.5)	0	38 (2.4)	1,350 (84.4)	210 (13.1)	1 (0.1)
70–74	769 (13.2)	0	0	439 (57.1)	315 (41.0)	15 (2.0)	1,081 (10.5)	0	0	558 (51.6)	506 (46.8)	17 (1.6)
All groups	5,822 (35.9)	571 (9.8)	2,082 (35.8)	2,616 (44.9)	537 (9.2)	16 (0.3)	10,309 (64.1)	1,388 (13.5)	4,008 (38.9)	4,141 (40.2)	754 (7.3)	18 (0.2)

**Figure 2:**
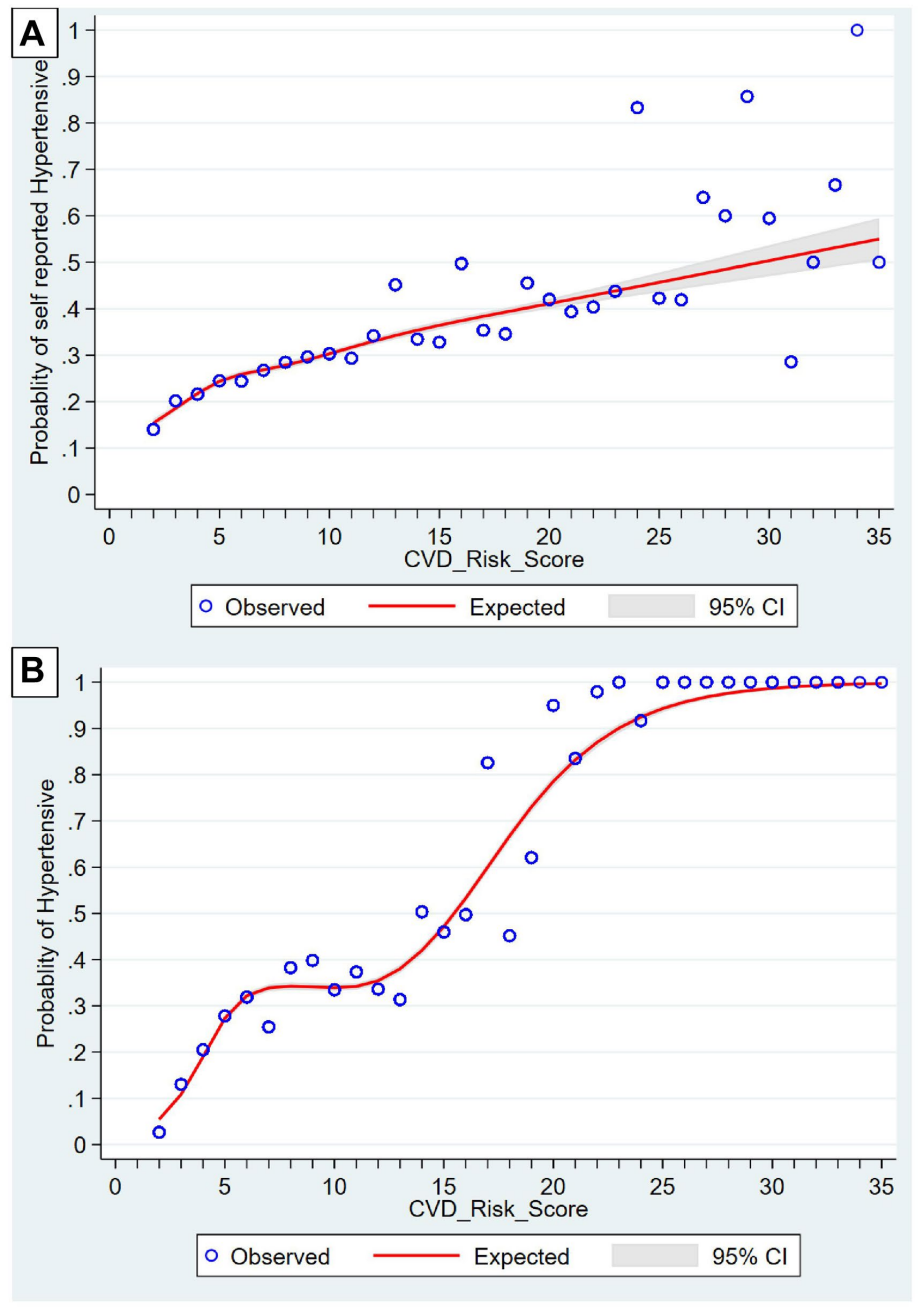
Distribution of CVD risk score as per **(A)** Self-reported hypertensive and **(B)** actual hypertensives using restrictive cubic splines.

Figure 2 describes the graphical representation of Distribution of CVD risk score as per (A) self-reported hypertensive and (B) Actual hypertensive using restricted cubic splines. Both graphs showed upward trend. In the initial 0 to 10 score, self-reported hypertensives were distributed symmetrically. With increase in the scores later, the distribution was less steep and was more scattered from the expected line (A). For actual hypertension, as defined by their blood pressure level, the initial 0 to 5 score, the trend was upward which followed a gentle plateau from 6 to 12; later following a steep curve, hitting a plateau at 30: overall following a sigmoid pattern. The distribution of hypertensives was along the line and less dispersed (B).

As shown in Table 4, the urban residents had 12% lower odds of being in a higher CVD risk score category rather than a combined lower risk score category, compared to the rural population. When categorised by education status, individuals with less than primary education had 1.21 times higher odds of being in a higher CVD risk score category, compared to those with no schooling. However, those with middle-school, secondary school, higher secondary and diploma, and graduate and higher education had lower odds of being in a higher CVD risk score category. Compared to married individuals, unmarried individuals had 14% less odds of being in a higher category group, while widowed/separated/divorced individuals had 1.93 times higher odds for the same. When categorised by MPCE quintiles, the richer and richest quintiles had 6 and 14% lower odds, respectively of being in a higher CVD risk score category, compared to the poorest quintile. Those who exercised regularly had 32% lower odds of being in a higher risk score category than those who did not exercise regularly. Individuals with a known history of diabetes had 1.69 times higher odds of being in a higher CVD risk score category when compared to individuals without these conditions (Table 4).

**Table 4:** Univariate and multivariable analysis between socio-demographic and disease factors with CVD risk score.

Variable	Total population, *N* (%)	Crude odds ratio (95% CI)	*p*-value	Adjusted odds ratio (95% CI)	*p*-value
*Socio-demographic factors*					
Residence					
Rural	33,951 (66.1)	Ref		Ref	
Urban	17,373 (33.9)	0.91 (0.88–0.94)	< 0.001	0.88 (0.85–0.91)	< 0.001
Education					
No schooling	23,681 (46.1)	Ref		Ref	
Less than primary (till 4)	5,841 (11.4)	1.14 (1.08–1.20)	< 0.001	1.21 (1.15–1.28)	< 0.001
Primary completed (5–7)	6,959 (13.6)	0.91 (0.87–0.96)	< 0.001	0.98 (0.94–1.03)	0.513
Middle completed (8–9)	5,136 (10.0)	0.77 (0.72–0.81)	< 0.001	0.86 (0.82–0.91)	< 0.001
Secondary school (10–11)	4,658 (9.1)	0.83 (0.79–0.88)	< 0.001	0.95 (0.89–1.00)	0.068
Higher Secondary	2,255 (4.4)	0.66 (0.61–0.71)	< 0.001	0.76 (0.70–0.83)	< 0.001
Diploma and graduate and above	2,794 (5.4)	0.70 (0.65–0.75)	< 0.001	0.85 (0.78–0.92)	< 0.001
Marital status					
Unmarried	651 (1.3)	0.82 (0.71–0.94)	0.006	0.86 (0.74–0.99)	0.038
Married/ in live -in	40,420 (78.)	Ref		Ref	
Widow/ separated/ divorced	10,252 (20.0)	2.00 (1.92–2.08)	<0.001	1.93 (1.85–2.00)	< 0.001
MPCE quintile					
Poorest	10,286 (20.0)	Ref		Ref	
Poorer	10,460 (20.4)	0.98 (0.93–1.03)	0.462	1.00 (0.95–1.05)	0.921
Middle	10,372 (20.2)	0.94 (0.89–0.98)	0.010	0.96 (0.91–1.01)	0.084
Richer	10,264 (20.0)	0.91 (0.86–0.96)	< 0.001	0.94 (0.89–0.99)	0.014
Richest	9,942 (19.4)	0.82 (0.78–0.87)	< 0.001	0.86 (0.81–0.90)	< 0.001
*Disease factors*					
Regular exercise					
No	13,298 (25.9)	Ref		Ref	
Yes	38,026 (74.1)	0.67 (0.64–0.69)	< 0.001	0.68 (0.66–0.71)	< 0.001
Known diabetes					
No	45,262 (88.2)	Ref		Ref	
Yes	6,046 (11.8)	1.59 (1.52–1.67)	< 0.001	1.69 (1.60–1.77)	< 0.001
Known hypercholesterolemia					
No	49,804 (97.1)	Ref		Ref	
Yes	1,514 (2.9)	1.03 (0.94–1.14)	0.459	1.00 (0.91–1.10)	0.943

## Discussion

This study estimated the 10-year risk for fatal and non-fatal cardiovascular disease using the 2019 WHO Non-laboratory-based risk prediction tool in the Indian population of ≥45-years. It was observed that two-thirds of the population had a 10-year CVD risk of <10%, and only a minority had a risk of ≥20%. The results are comparable to a study conducted in rural Andhra Pradesh, where 83.1% of the total population had a risk score of <10%, although the proportion of the population with a high CVD risk score was much higher in that population, i.e., 7.8% (21). The CVD risk score in our population was seen to be comparable to studies in the neighbouring countries. In a hospital-based cross-sectional study in Nepal, two-thirds of the participants, including 80% of females and half of the males had a 10-year CVD risk score of <10% (22). A cross-sectional analysis of individuals in rural and urban Bangladesh showed that half of the population had a risk of <5%, with 15% of the population at risk of >10%. This included almost one-fifth of the male population and 8% of the female population (23).

While the WHO CVD Risk Score is commonly used, other risk scores have been utilized for Indian and other populations in LMICs. These include the Framingham Risk Score, the Globorisk and the older WHO/ ISH classification. A study using nationally representative data of individuals aged 30 to 74 in India showed that sizable number of females and males had a high risk of CVD when classified using the WHO/ISH risk score (24). In male individuals from Tamil Nadu, it was found that majority of the urban and of the rural population had a 10-year risk of <10%, using the Framingham Risk Score (25).

For self-reported hypertension, the distribution was seen to be more scattered from the expected line at a higher CVD risk score. This suggests that there is more uncertainty at this level of CVD risk score, implying that these may not be aware of their true hypertension status, i.e., those with a higher CVD risk score have less knowledge of their hypertension status.

In this population, while three-fourths of individuals aged 45–49 years had a CVD Risk Score of <5%, none of those aged 60 years and above had this risk. The risk of ≥30% was seen only in individuals 65 years and above. With ageing, the risk of CVD escalates due to changes in the heart caused by myocardial deterioration and degeneration (26). As expected, both males and females were shown to have an increased risk for CVD with increasing age.

The risk was higher among males compared to females, as observed in this study and in a national representative survey of individuals aged less than 49 years in the country, and in the study conducted in Andhra Pradesh (21, 27). The difference between sexes can be attributed to the presence of sex hormones and other sex chromosome complement. Pre-menopausal women are known to have a lower blood pressure and associated cardiovascular diseases due to modulation provided by sex hormones (28). Oestrogen has a cardioprotective effect due to its impact on the renin-angiotensin-aldosterone and the endothelin systems (28). However, these protections are lost in the post-menopausal women. In this study, the post-menopausal women too had a lower risk, which may partly be attributed to a much higher prevalence of tobacco use in males compared to females. Tobacco causes increased sympathetic activation leading to an increase in blood pressure. Endothelial dysfunction and reduced endothelial repair caused by smoking lead to an atherosclerotic environment in the vessels (29). Tobacco smokers are more likely to experience an acute cardiovascular event at a younger age and earlier in the course of their disease (30). In India, 28.6% of the population, including 42.4% of men and 14.2% of women, use tobacco in some form (31).

In this study, the urban population was observed to have a lower risk for developing cardiovascular disease compared to the rural population. Additionally, individuals in the richest quintile and those with higher education levels were seen to have a lower risk. Non-communicable diseases were previously more commonly seen in the affluent population. Lately, non-communicable diseases are seen to be prevalent among the poor in the Low- and Middle-Income Countries (32). This may be because of a higher prevalence of risk factors including tobacco use, alcohol consumption and an unhealthy diet consisting of fewer fruits, vegetables, fish, and fibre, when compared to their wealthier counterparts (33). Conversely, the presence of chronic diseases itself may drive them into poverty (32).

Physical activity decreases the risk of and mortality due to cardiovascular disease. It also reduces the risk of developing risk factors for CVD, including hypertension, diabetes and high cholesterol levels (34). Those without regular exercise and those with a history of diabetes were shown to have a higher risk of developing cardiovascular disease. The inflammatory state in obesity and diabetes and its effect on blood pressure, cholesterol, and sugar levels causes an increase in cardiovascular risk in these individuals (35). However, this study did not find any significant association between increased cholesterol levels and the risk of CVD. Excluding the individuals with known cardiovascular disease may be the reason of the non-significant association observed in this study.

This study has also categorised individuals into those with and without a known diagnosis of hypertension. In the population with blood pressure above the range to qualify as hypertension, two-thirds of the population did not have a diagnosis of hypertension. However, the CVD Risk did not differ significantly between these individuals. Additionally, hypertensive individuals who had their blood pressure under control had a similar risk as those without known hypertension in this study and a nationally representative survey (27). Management of a person should not just stop at controlling blood pressure; managing other factors would be equally important. This also indicates the importance of managing hypertension (27). This underscores the importance of regular health check-ups and maintaining a healthy lifestyle.

Some limitations do exist in this study. Since, it is a cross-sectional survey, causality between outcome and independent variables cannot be established. Since the LASI survey involved self-reported questions pertaining to CVD-risk, therefore chances of recall bias and social desirability bias cannot be neglected. These biases due to self-reported nature may result in under reporting or sometimes over reporting of the results (36). Despite these limitations, the present study has its own unique strengths. The large and representative sample in the survey enhances its generalizability to the Indian population. The standard methodology used in LASI and in the assessment of CVD risk score ensures consistency and reliability. Given that the same population will be followed up in the future waves of LASI, the study offers excellent replicability for the future research on CVD risk assessment. Thus, the study would be helpful for understanding and managing cardiovascular risk in India.

### Policy implications

The World Health Organisation (WHO) has set worldwide objectives with respect to the Global Monitoring Framework for the control of non-communicable diseases (NCDs) especially in context of Low- and middle-income countries (LMICs) like India. Eighty percent of the country’s basic affordable technologies and critical medications that are required to treat severe NCDs, including CVDs, must be readily available in both rural and urban regions (37). Weak healthcare infrastructure and limited access to CVD treatment are major public health concerns. Adequate regulations ought to tackle the possibility of both under and overtreatment, since it is quite expensive for the patient and the healthcare system. The substantial proportion of adults with a low 10-year CVD risk (<10%) underscores the possibility of lowering CVD risk by means of population-wide public health policies and the provision of easily available preventive interventions. But care should be taken to make sure that risk stratification techniques aren’t applied in unsuitable clinical situations, including those with severely uncontrolled hypertension (160/100 mm Hg) (24).

The sigmoid pattern in actual hypertensives highlights the need for early detection and initiation of treatments. Government of India has implemented population-based screening (PBS) as a part of implementation of comprehensive healthcare under National Program for Prevention and Control of Non-Communicable Diseases (NP-NCD) targeting population aged 30 years and above for screening for hypertension, diabetes, common cancers (breast, cervical and oral) implemented by trained frontline health professionals (ANM: Auxiliary Nurse Midwife; ASHA: Accredited Social Health Activist) (38). Camp-based screenings are practiced at regular intervals in the Indian community settings. Medical Officers at nearby Primary Health Centres (PHCs) receive suspicious patients and refer them for a second examination in the higher centres. The program seeks to lower the burden of NCDs by allowing early detection and prompt care (39). We recommend to include evaluation of CVD risk score in the same approach. By early detection, the treatment can be implemented at national and subnational levels especially in geographically unapproachable population.

## Conclusion

This study reveals that while the majority of the Indian population has a low risk of CVD, a small but significant proportion have a high risk. It also highlights the disparities among the different socio-economic and educational groups, with a shifting of the burden to the poorer population. It also emphasizes the protective role of physical activity against cardiovascular disease, suggesting the need for initiatives to encourage regular exercise. A large proportion of individuals with blood pressure in the levels of hypertension were undiagnosed but had a similar risk of developing cardiovascular disease. These findings highlight the importance of promoting regular health check-ups for early detection and management of hypertension.

## Data availability statement

The original contributions presented in the study are included in the article/Supplementary material, further inquiries can be directed to the corresponding authors.

## Ethics statement

The studies involving humans were approved by the original LASI study reports which were in compliance with research ethics. Informed written consent was obtained from the participants. Ethical clearance was permitted by the Indian Council of Medical Research (ICMR) and each collaborating bodies. Current study is based on secondary data analysis of Longitudinal Ageing Study in India (LASI) Wave- 1, India. This study was carried out in accordance with the Helsinki Declaration Principles (19). The studies were conducted in accordance with the local legislation and institutional requirements. The participants provided their written informed consent to participate in this study. Written informed consent was obtained from the individual(s) for the publication of any potentially identifiable images or data included in this article.

## Author contributions

AM: Conceptualization, Data curation, Formal analysis, Investigation, Methodology, Project administration, Resources, Software, Supervision, Validation, Visualization, Writing – original draft, Writing – review & editing. PH: Conceptualization, Data curation, Formal analysis, Investigation, Methodology, Project administration, Resources, Software, Supervision, Validation, Visualization, Writing – original draft, Writing – review & editing. AB: Writing – original draft, Writing – review & editing. KG: Writing – original draft, Writing – review & editing. SP: Writing – original draft, Writing – review & editing. KA: Writing – original draft, Writing – review & editing. DS: Conceptualization, Data curation, Investigation, Methodology, Software, Supervision, Writing – original draft, Writing – review & editing. TK: Conceptualization, Data curation, Formal analysis, Investigation, Methodology, Project administration, Resources, Software, Supervision, Validation, Visualization, Writing – original draft, Writing – review & editing.
