# Supplementary material for: Cardiovascular risk assessment using non-laboratory based WHO CVD risk prediction chart with respect to hypertension status among older Indian adults: insights from nationally representative survey
Source: Front Public Health. 2024 Sep 5;12:1407918. doi: 10.3389/fpubh.2024.1407918 (PMC11410575; doi:10.3389/fpubh.2024.1407918)
Supplement: Supplementary file 1 [file Table_1.docx]

**Cardiovascular Risk Assessment Using Non-Laboratory Based WHO CVD Risk Prediction Chart with Respect to Hypertension Status among Older Indian Adults: Insights from Nationally Representative Survey**

**Supplementary**

Supplementary Table 1: Variables used for development of CVD Risk Score

| S. No | Variables | Categories |
| --- | --- | --- |
|  | Age | 40-44 |
|  |  | 45-49 |
|  |  | 50-54 |
|  |  | 55-59 |
|  |  | 60-64 |
|  |  | 65-69 |
|  |  | 70-74 |
|  | Sex | Women |
|  |  | Men |
|  | Smoking Status | Non-smoker |
|  |  | Smoker |
|  | BMI category | < 20 |
|  |  | 20-24 |
|  |  | 25-29 |
|  |  | 30-35 |
|  |  | ≥ 35 |
|  | Systolic Blood Pressure | < 120 |
|  |  | 120-139 |
|  |  | 140-159 |
|  |  | 160-179 |
|  |  | ≥ 180 |

Supplementary Table 2: Socio-demographic characteristics according to HTN status

| **Variables** | **Total population** | **Female** | | | **Male** | | |
| --- | --- | --- | --- | --- | --- | --- | --- |
|  |  | **Total n (%),**  **27,906 (54.4)** | **High blood pressure (%) 8,424 (30.2%)** | **Self-reported Hypertensive**  **(%)**  **8,325 (29.8%)** | **Total n (%), 23,418 (45.6)** | **High blood pressure (%), 7729**  **(33.0%)** | **Self-reported Hypertensive (%)**  **4,976 (21.2%)** |
| **Age-group (years)** | | | | | | | |
| 45-49 | 11,861 (23.1) | 6,613 (23.7) | 1,397 (21.1) | 1,404 (21.2) | 5,248 (22.4) | 1,522 (29.0) | 749 (14.3) |
| 50-54 | 9,686 (18.9) | 5,306 (19.0) | 1,442 (27.2) | 1,426 (26.9) | 4,380 (18.7) | 1,344 (30.7) | 778 (17.8) |
| 55-59 | 8,778 (17.1) | 4,927 (17.7) | 1,490 (30.2) | 1,485 (30.2) | 3,851 (16.4) | 1,278 (33.2) | 807 (21.0) |
| 60-64 | 8,785 (17.1) | 4,815 (17.2) | 1,670 (34.7) | 1,614 (33.5) | 3,970 (17.0) | 1,379 (34.7) | 909 (23.0) |
| 65-69 | 7,476 (14.6) | 3,857 (13.8) | 1,458 (37.8) | 1,448 (37.5) | 3,619 (15.4) | 1,303 (36.0) | 1,043 (28.8) |
| 70-74 | 4,738 (9.2) | 2,388 (8.6) | 967 (40.5) | 948 (39.7) | 2,350 (10.0) | 883 (37.6) | 690 (29.4) |
| **Residence** | | | | | | | |
| Urban | 17,373 (33.9) | 9,641 (34.6) | 3,153 (32.7) | 3,633 (37.7) | 7,732 (33.0) | 2,869 (37.1) | 2,174 (28.1) |
| Rural | 33,951 (66.1) | 18,265 (65.4) | 5,271 (28.9) | 4,692 (25.7) | 15,686 (67.0) | 4,840 (30.9) | 2,802 (17.9) |
| **Education** | | | | | | | |
| No schooling | 23,681 (46.1) | 16,599 (59.5) | 4,995 (30.1) | 4,438 (26.7) | 7,082 (30.2) | 2,101 (39.7) | 1,152 (16.3) |
| Less than primary (till 4) | 5,841 (11.4) | 2,736 (9.8) | 859 (31.4) | 919 (33.6) | 3,105 (13.3) | 1,011 (32.6) | 555 (17.9) |
| Primary completed (5-7) | 6,959 (13.6) | 3,255 (11.7) | 1,002 (30.8) | 1,132 (34.8) | 3,704 (15.8) | 1,232 (33.3) | 795 (21.5) |
| Middle completed (8-9) | 5,136 (10.0) | 2,021 (7.2) | 614 (30.4) | 687 (34.0) | 3,115 (13.3) | 1,055 (33.9) | 644 (20.7) |
| Secondary school (10-11) | 4,658 (9.1) | 1,690 (6.1) | 521 (30.8) | 612 (36.2) | 2,968 (12.7) | 1,094 (36.9) | 796 (26.8) |
| Higher secondary | 2,255 (4.4) | 741 (2.7) | 210 (28.3) | 248 (33.5) | 1,514 (6.5) | 541 (35.7) | 426 (28.2) |
| Diploma and graduate and above | 2,794 (5.4) | 864 (3.1) | 223 (25.8) | 289 (33.5) | 1,930 (8.2) | 675 (35.0) | 608 (31.5) |
| **Marital Status** | | | | | | | |
| Unmarried | 651 (1.3) | 295 (1.1) | 94 (31.9) | 77 (26.1) | 356 (1.5) | 102 (28.7) | 56 (15.7) |
| Married/ in live -in | 40,420 (78.) | 19,205 (68.8) | 5,264 (27.4) | 5,299 (27.6) | 21,215 (90.6) | 6,889 (32.5) | 4,492 (21.2) |
| Widow/ separated/ divorced | 10,252 (20.0) | 8,406 (30.1) | 3,066 (36.5) | 2,949 (35.1) | 1,846 (7.9) | 717 (38.8) | 427 (23.1) |
| **MPCE Quintile** | | | | | | | |
| Poorest | 10,286 (20.0) | 5,581 (20.0) | 1,721 (30.8) | 1,296 (23.3) | 4,705 (20.1) | 1,480 (31.5) | 732 (15.6) |
| Poorer | 10,460 (20.4) | 5,728 (20.5) | 1,725 (30.1) | 1,556 (27.2) | 4,732 (20.2) | 1,504 (31.8) | 876 (18.5) |
| Middle | 10,372 (20.2) | 5,631 (20.2) | 1,711 (30.4) | 1,719 (30.5) | 4,741 (20.3) | 1,581 (33.4) | 973 (20.5) |
| Richer | 10,264 (20.0) | 5,543 (19.9) | 1,657 (30.0) | 1,852 (33.4) | 4,721 (20.2) | 1,596 (33.8) | 1,137 (24.1) |
| Richest | 9,942 (19.4) | 5,423 (19.4) | 1,610 (29.7) | 1,902 (35.1) | 4,519 (19.3) | 1,548 (34.3) | 1,258 (27.9) |
| **Regular Exercise** | | | | | | | |
| No | 13,298 (25.9) | 6,305 (22.6) | 1,975 (31.3) | 2,110 (33.5) | 6,993 (29.9) | 2,432 (34.8) | 1,696 (24.3) |
| Yes | 38,026 (74.1) | 21,601 (77.4) | 6,449 (29.9) | 6,215 (28.8) | 16,425 (70.1) | 5,277 (32.1) | 3,280 (20.0) |
| **Smoking Status** | | | | | | | |
| Non-smoker | 32,667 (63.7) | 22,464 (80.5) | 6,738 (30.0) | 6,850 (30.5) | 10,203 (43.6) | 3,604 (35.3) | 2,601 (25.6) |
| Smoker | 18,657 (36.3) | 5,442 (19.5) | 1,686 (31.0) | 1,475 (27.1) | 13,215 (56.4) | 4,105 (31.1) | 2,375 (18.0) |
| **BMI category** | | | | | | | |
| < 20 | 14,906 (29.0) | 7,650 (27.4) | 1,775 (23.2) | 1,359 (17.8) | 7,256 (31.0) | 1,662 (22.9) | 918 (12.7) |
| 20-24 | 21,025 (41.0) | 10,576 (37.9) | 3,095 (29.3) | 2,863 (27.1) | 10,449 (44.6) | 3,547 (34.0) | 2,148 (20.6) |
| 25-29 | 11,606 (22.6) | 6,817 (24.4) | 2,471 (36.3) | 2,704 (39.7) | 4,789 (20.5) | 2,058 (43.0) | 1,540 (32.2) |
| 30-35 | 3,137 (6.1) | 2,304 (8.3) | 882 (38.3) | 1,092 (47.4) | 833 (3.6) | 399 (47.9) | 334 (40.1) |
| ≥ 35 | 650 (1.3) | 559 (2.0) | 201 (36.0) | 307 (54.9) | 91 (0.4) | 43 (47.3) | 36 (39.7) |
| **Systolic Blood Pressure** | | | | | | | |
| < 120 | 18,725 (36.5) | 10,592 (38.0) | 94 (0.9) | 2,122 (20.1) | 8,133 (34.7) | 112 (1.4) | 1,041 (12.8) |
| 120-139 | 20,112 (39.2) | 10,432 (37.4) | 1,448 (13.9) | 3,221 (30.9) | 9,680 (41.3) | 1,992 (20.6) | 2,000 (207) |
| 140-159 | 9,139 (17.8) | 4,941 (17.7) | 100.0 | 2,015 (40.8) | 4,198 (17.9) | 100.0 | 1,351 (32.9) |
| 160-179 | 3,160 (6.2) | 1,824 (6.5) | 100.0 | 914 (50.1) | 1,336 (5.7) | 100.0 | 559 (41.8) |
| ≥ 180 | 188 (0.4) | 117 (0.4) | 100.0 | 53 (45.3) | 71 (0.3) | 100.0 | 1,041 (12.8) |
| **Known Diabetes** | | | | | | | |
| No | 45,262 (88.2) | 24,618 (88.2) | 7,146 (29.0) | 6,276 (25.6) | 20,644 (88.2) | 6,578 (31.9) | 3,511 (17.0) |
| Yes | 6,046 (11.8) | 3,281 (11.8) | 1,277 (38.9) | 2,048 (62.4) | 2,765 (11.8) | 1,130 (40.9) | 1,465 (53.0) |
| **Known Hypercholesterolemia** | | | | | | | |
| No | 49,804 (97.1) | 26,951 (96.6) | 8,059 (29.9) | 7,683 (28.5) | 22,853 (97.6) | 7,501 (32.8) | 4,646 (20.3) |
| Yes | 1,514 (2.9) | 950 (3.4) | 364 (38.3) | 642 (67.6) | 564 (2.4) | 208 (36.9) | 330 (58.5) |

Supplementary Table 3: Distribution of the risk factors used for development of non-lab-based CVD risk score in the study population.

| **Categories** | **Total (N= 53,804)** | **Female (N= 29,022), 53.9%** | **Male (N= 24,782), 46.1%** |
| --- | --- | --- | --- |
| **Age** | | | |
| 45-49 | 11,861 (23.1) | 6,613 (23.7) | 5,248 (22.4) |
| 50-54 | 9,686 (18.9) | 5,306 (19.0) | 4,380 (18.7) |
| 55-59 | 8,778 (17.1) | 4,927 (17.7) | 3,851 (16.4) |
| 60-64 | 8,785 (17.1) | 4,815 (17.2) | 3,970 (17.0) |
| 65-69 | 7,476 (14.6) | 3,857 (13.8) | 3,619 (15.4) |
| 70-74 | 4,738 (9.2) | 2,388 (8.6) | 2,350 (10.0) |
| **Smoking Status** | | | |
| Non-smoker | 32,667 (63.7) | 22,464 (80.5) | 10,203 (43.6) |
| Smoker | 18,657 (36.3) | 5,442 (19.5) | 13,215 (56.4) |
| **BMI category** | | | |
| < 20 | 15,381 (28.6) | 7,650 (27.4) | 7,256 (31.0) |
| 20-24 | 22,041 (41.0) | 10,576 (37.9) | 10,449 (44.6) |
| 25-29 | 12,346 (23.0) | 6,817 (24.4) | 4,789 (20.5) |
| 30-35 | 3,337 (6.2) | 2,304 (8.3) | 833 (3.6) |
| ≥ 35 | 699 (1.3) | 559 (2.0) | 91 (0.4) |
| **Systolic Blood Pressure** | | | |
| < 120 | 19,467 (36.2) | 10,904 (37.6) | 8,563 (34.6) |
| 120-139 | 21,109 (39.2) | 10,885 (37.5) | 10,224 (41.3) |
| 140-159 | 9,653 (17.9) | 5,172 (17.8) | 4,481 (18.1) |
| 160-179 | 3,375 (6.3) | 1,941 (6.7) | 1,434 (5.8) |
| ≥ 180 | 200 (0.4) | 120 (0.4) | 80 (0.3) |

Supplementary Table 4: CVD risk among male and female participants in the different age groups.

| **Variables** | **Female** | | | | | | | **Male** | | | | | | |
| --- | --- | --- | --- | --- | --- | --- | --- | --- | --- | --- | --- | --- | --- | --- |
|  | **Total** | **<5%, (%)** | **5-< 10%, (%)** | **10-< 20%, (%)** | **20-< 30%, (%)** | **>30%, (%)** | **Total** | | **<5%, (%)** | **5-< 10%, (%)** | **10-< 20%, (%)** | **20-< 30%, (%)** | **>30%, (%)** |  |
| **Age-group (years)** |  |  |  |  |  |  |  |  |  |  |  |  |  |  |
| 45-49 | 6,613 (23.7) | 5,840 (88.3) | 737 (11.1) | 36 (0.5) | 0 | 0 | 5,248 (22.4) | | 3,325 (63.4) | 1,843 (35.1) | 80 (1.5) | 0 | 0 |  |
| 50-54 | 5,306 (19.0) | 3,496 (65.9) | 1,665 (31.4) | 145 (2.7) | 0 | 0 | 4,380 (18.7) | | 1,159 (26.5) | 2,806 (64.1) | 414 (9.5) | 1 (0.0) | 0 |  |
| 55-59 | 4,927 (17.7) | 1,379 (28.0) | 3,230 (65.6) | 317 (6.4) | 1 (0.0) | 0 | 3,851 (16.4) | | 165 (4.3) | 2,450 (63.6) | 1,201 (31.2) | 35 (0.9) | 0 |  |
| 60-64 | 4,815 (17.3) | 0 | 3,747 (77.8) | 1,057 (22.0) | 11 (0.2) | 0 | 3,970 (17.0) | | 0 | 1,591 (40.1) | 2,335 (58.8) | 44 (1.1) | 0 |  |
| 65-69 | 3,857 (13.8) | 0 | 1,548 (40.1) | 2,203 (57.1) | 106 (2.8) | 0 | 3,619 (15.5) | | 0 | 326 (9.0) | 3,017 (83.4) | 274 (7.6) | 2 (0.1) |  |
| 70-74 | 2,388 (8.6) | 0 | 0 | 2,131 (89.2) | 254 (10.6) | 3 (0.1) | 2,350 (10.0) | | 0 | 0 | 1,686 (71.7) | 635 (27.0) | 29 (1.2) |  |

Supplementary Table 5: CVD risk among the participants with controlled blood pressure, categorised by sexes.

| **Female** | | | | | | | | | | | | |
| --- | --- | --- | --- | --- | --- | --- | --- | --- | --- | --- | --- | --- |
| **45-49** | 887 (18.2) | 815 (91.9) | 72 (8.1) | 0 | 0 | 0 | 4,328 (29.6) | 4,044 (93.4) | 284 (6.6) | 0 | 0 | 0 |
| **50-54** | 881 (18.1) | 757 (85.9) | 124 (14.1) | 0 | 0 | 0 | 2,982 (20.4) | 2,432 (81.6) | 550 (18.4) | 0 | 0 | 0 |
| **55-59** | 895 (18.4) | 283 (31.6) | 581 (64.9) | 31 (3.5) | 0 | 0 | 2,541 (17.4) | 1,083 (42.6) | 1,413 (55.6) | 45 (1.8) | 0 | 0 |
| **60-64** | 922 (18.9) | 0 | 800 (86.8) | 122 (13.2) | 0 | 0 | 2,221 (15.2) | 0 | 1,966 (88.5) | 255 (11.5) | 0 | 0 |
| **65-69** | 779 (16.0) | 0 | 419 (53.8) | 360 (46.2) | 0 | 0 | 1,620 (11.1) | 0 | 1,077 (66.5) | 543 (33.5) | 0 | 0 |
| **70-74** | 503 (10.3) | 0 | 0 | 502 (99.8) | 1 (0.2) | 0 | 917 (6.3) | 0 | 0 | 915 (99.8) | 2 (0.2) | 0 |
| **All groups** | 4,867 | 1,855 (38.1) | 1.996 (41.0) | 1,015 (20.9) | 1 (0.0) | 0 | 14,609 | 7,559 (51.7) | 5,290 (36.2) | 1,758 (12.0) | 2 (0.0) | 0 |
| **Male** | | | | | | | | | | | | |
| **45-49** | 400 (15.3) | 299 (74.8) | 101 (25.2) | 0 | 0 | 0 | 3,323 (25.4) | 2,499 (75.2) | 819 (24.7) | 5 (0.1) | 0 | 0 |
| **50-54** | 407 (15.6) | 140 (34.4) | 263 (64.6) | 4 (1.0) | 0 | 0 | 2,629 (20.1) | 884 (33.6) | 1,730 (65.8) | 15 (0.6) | 0 | 0 |
| **55-59** | 406 (15.5) | 13 (3.2) | 307 (75.6) | 86 (21.2) | 0 | 0 | 2,166 (16.6) | 152 (7.0) | 1,602 (74.0) | 412 (19.0) | 0 | 0 |
| **60-64** | 482 (15.6) | 0 | 270 (56.0) | 212 (44.0) | 0 | 0 | 2,107 (16.1) | 0 | 1,145 (54.3) | 962 (45.7) | 0 | 0 |
| **65-69** | 551 (21.1) | 0 | 55 (10.0) | 494 (89.7) | 2 (0.3) | 0 | 1,765 (16.5) | 0 | 269 (15.2) | 1,496 (84.8) | 0 | 0 |
| **70-74** | 366 (14.0) | 0 | 0 | 338 (92.4) | 28 (7.6) | 0 | 1,101 (8.4) | 0 | 0 | 1,064 (96.6) | 37 (3.4) | 0 |
| **All groups** | 2,612 | 499 (16.2) | 1,152 (37.5) | 1,388 (45.1) | 37 (1.2) | 0 | 13,091 | 3,535 (27.0) | 5,565 (42.5) | 3,954 (30.2) | 37 (0.3) | 0 |

Supplementary Table 6: CVD Risk Score among participants with uncontrolled blood pressure, categorised by sexes.

| **Female** | | | | | | | | | | | | |
| --- | --- | --- | --- | --- | --- | --- | --- | --- | --- | --- | --- | --- |
| **45-49** | 517 (15.0) | 342 (66.2) | 155 (30.0) | 20 (3.8) | 0 | 0 | 879 (17.7) | 638 (72.6) | 225 (25.6) | 16 (1.8) | 0 | 0 |
| **50-54** | 545 (15.8) | 93 (17.1) | 387 (71.0) | 65 (11.9) | 0 | 0 | 897 (18.1) | 214 (23.9) | 603 (67.2) | 80 (8.9) | 0 | 0 |
| **55-59** | 590 (117.1) | 3 (0.5) | 490 (83.1) | 96 (16.3) | 1 (0.2) | 0 | 900 (18.1) | 9 (1.0) | 746 (82.9) | 145 (16.1) | 0 | 0 |
| **60-64** | 692 (20.0) | 0 | 394 (56.9) | 290 (41.9) | 8 (1.2) | 0 | 978 (19.7) | 0 | 586 (59.9) | 389 (39.8) | 3 (0.3) | 0 |
| **65-69** | 669 (19.4) | 0 | 16 (2.4) | 606 (90.6) | 47 (7.0) | 0 | 789 (15.9) | 0 | 36 (4.6) | 694 (88.0) | 59 (7.5) | 0 |
| **70-74** | 445 (12.9) | 0 | 0 | 338 (76.0) | 105 (23.6) | 2 (0.4) | 522 (10.5) | 0 | 0 | 375 (71.8) | 146 (28.0) | 1 (0.2) |
| **All groups** | 3,458 | 438 (13.7) | 1,442 (41.7) | 1,415 (40.9) | 161 (4.7) | 2 (0.1) | 4,965 | 861 (17.3) | 2,196 (44.2) | 1,699 (34.2) | 208 (4.2) | 1 (0.0) |
| **Male** | | | | | | | | | | | | |
| **45-49** | 349 (14.8) | 107 (30.7) | 214 (61.3) | 28 (8.0) | 0 | 0 | 1,173 (22.0) | 418 (35.6) | 708 (60.4) | 47 (4.0) | 0 | 0 |
| **50-54** | 371 (15.7) | 26 (7.0) | 221 (59.6) | 123 (33.2) | 1 (0.3) | 0 | 973 (18.2) | 109 (11.2) | 592 (60.8) | 272 (28.0) | 0 | 0 |
| **55-59** | 401 (17.0) | 0 | 168 (41.9) | 212 (52.9) | 21 (5.2) | 0 | 877 (16.4) | 0 | 373 (42.5) | 490 (55.9) | 14 (1.6) | 0 |
| **60-64** | 427 (18.1) | 0 | 37 (8.7) | 367 (86.0) | 23 (5.3) | 0 | 952 (17.8) | 0 | 137 (14.4) | 794 (83.4) | 21 (2.2) | 0 |
| **65-69** | 492 (20.8) | 0 | 0 | 370 (75.2) | 121 (24.6) | 1 (0.2) | 810 (15.2) | 0 | 2 (.03) | 656 (81.0) | 151 (18.6) | 1 (0.1) |
| **70-74** | 324 (13.7) | 0 | 0 | 101 (31.2) | 210 (64.8) | 13 (4.0) | 559 (10.5) | 0 | 0 | 183 (32.7) | 360 (64.4) | 16 (2.9) |
| **All groups** | 2,364 | 133 (5.6) | 640 (27.1) | 1,201 (50.8) | 376 (15.9) | 14 (0.6) | 5,344 | 527 (9.9) | 1,812 (33.9) | 2,442 (45.7) | 546 (10.2) | 17 (0.3) |
