# Supplementary material for: Cardiovascular risk assessment using non-laboratory based WHO CVD risk prediction chart with respect to hypertension status among older Indian adults: insights from nationally representative survey
Source: Front Public Health. 2024 Sep 5;12:1407918. doi: 10.3389/fpubh.2024.1407918 (PMC11410575; doi:10.3389/fpubh.2024.1407918)
Supplement: Supplementary file 2 [file Data_Sheet_1.PDF]

```
*Pre-requisites:

*Sex as a byte variable coded as categories: (1- Male,  2 - Female)
*Smoking_status as a byte variable coded as categories: (1- Smoker,  2- Non Smoker)
*Age_cat as byte variable codes as categories: (1- 45 to 49 age group, 2- 50 to 54 age group, 3-55 to 59 age group, 4- 60 to 64 age group, 5- 65 to 69 age group, 6- 70 to 74 age group
*BMI as string variable with categories: <20, 20-24, 25-29, 30-35, >=35
*SBP as string variable with categories: <120, 120-139, 140-159, 160-179, >=180

* Generate a New Variable for CVD_Risk_Score.
gen CVD_Risk_Score=.

* Loop through each observation
forvalues i = 1/`=_N' {
    * Check the conditions for sex, smoking status, and age category
    display "Sex[`i'] = " Sex[`i'] " Smoking_status[`i'] = " Smoking_status[`i'] " Age_cat[`i'] = " Age_cat[`i'] " BMI[`i'] = " BMI[`i'] " SBP[`i'] = " SBP[`i'] " CVD_Risk_Score[`i'] = " CVD_Risk_Score[`i']
    *Starting with Sex - 1, Smoking status-2 and Age category-6
     if Sex[`i'] == 1 {
        if Smoking_status[`i'] == 2 {
            if Age_cat[`i'] == 6 {
        * Check the condition for BMI and update the CVD risk score based on systolic blood pressure
        if BMI[`i'] == "<20" {
            replace CVD_Risk_Score = 11 if SBP[`i'] == "<120" in `i'
            replace CVD_Risk_Score = 14 if SBP[`i'] == "120-139" in `i'
            replace CVD_Risk_Score = 17 if SBP[`i'] == "140-159" in `i'
            replace CVD_Risk_Score = 20 if SBP[`i'] == "160-179" in `i'
            replace CVD_Risk_Score = 24 if SBP[`i'] == ">=180" in `i'
        }
        else if BMI[`i'] == "20-24" {
            replace CVD_Risk_Score = 12 if SBP[`i'] == "<120" in `i'
            replace CVD_Risk_Score = 15 if SBP[`i'] == "120-139" in `i'
            replace CVD_Risk_Score = 18 if SBP[`i'] == "140-159" in `i'
            replace CVD_Risk_Score = 22 if SBP[`i'] == "160-179" in `i'
            replace CVD_Risk_Score = 26 if SBP[`i'] == ">=180" in `i'
        }
        else if BMI[`i'] == "25-29" {
            replace CVD_Risk_Score = 13 if SBP[`i'] == "<120" in `i'
            replace CVD_Risk_Score = 16 if SBP[`i'] == "120-139" in `i'
            replace CVD_Risk_Score = 19 if SBP[`i'] == "140-159" in `i'
            replace CVD_Risk_Score = 23 if SBP[`i'] == "160-179" in `i'
            replace CVD_Risk_Score = 28 if SBP[`i'] == ">=180" in `i'
        }
        else if BMI[`i'] == "30-35" {
            replace CVD_Risk_Score = 14 if SBP[`i'] == "<120" in `i'
            replace CVD_Risk_Score = 17 if SBP[`i'] == "120-139" in `i'
            replace CVD_Risk_Score = 21 if SBP[`i'] == "140-159" in `i'
            replace CVD_Risk_Score = 25 if SBP[`i'] == "160-179" in `i'
            replace CVD_Risk_Score = 30 if SBP[`i'] == ">=180" in `i'
        }
        else if BMI[`i'] == ">=35" {
            replace CVD_Risk_Score = 15 if SBP[`i'] == "<120" in `i'
            replace CVD_Risk_Score = 18 if SBP[`i'] == "120-139" in `i'
            replace CVD_Risk_Score = 22 if SBP[`i'] == "140-159" in `i'
            replace CVD_Risk_Score = 27 if SBP[`i'] == "160-179" in `i'
            replace CVD_Risk_Score = 32 if SBP[`i'] == ">=180" in `i'
        }
    }
    else if Age_cat[`i'] == 5 {
        * Check the condition for BMI and update the CVD risk score based on systolic blood pressure
        if BMI[`i'] == "<20" {
            replace CVD_Risk_Score = 8 if SBP[`i'] == "<120" in `i'
            replace CVD_Risk_Score = 10 if SBP[`i'] == "120-139" in `i'
            replace CVD_Risk_Score = 12 if SBP[`i'] == "140-159" in `i'
            replace CVD_Risk_Score = 15 if SBP[`i'] == "160-179" in `i'
```

```
            replace CVD_Risk_Score = 19 if SBP[`i'] == ">=180" in `i'
        }
        else if BMI[`i'] == "20-24" {
            replace CVD_Risk_Score = 9 if SBP[`i'] == "<120" in `i'
            replace CVD_Risk_Score = 11 if SBP[`i'] == "120-139" in `i'
            replace CVD_Risk_Score = 14 if SBP[`i'] == "140-159" in `i'
            replace CVD_Risk_Score = 17 if SBP[`i'] == "160-179" in `i'
            replace CVD_Risk_Score = 20 if SBP[`i'] == ">=180" in `i'
        }
        else if BMI[`i'] == "25-29" {
            replace CVD_Risk_Score = 10 if SBP[`i'] == "<120" in `i'
            replace CVD_Risk_Score = 12 if SBP[`i'] == "120-139" in `i'
            replace CVD_Risk_Score = 15 if SBP[`i'] == "140-159" in `i'
            replace CVD_Risk_Score = 18 if SBP[`i'] == "160-179" in `i'
            replace CVD_Risk_Score = 22 if SBP[`i'] == ">=180" in `i'
        }
        else if BMI[`i'] == "30-35" {
            replace CVD_Risk_Score = 11 if SBP[`i'] == "<120" in `i'
            replace CVD_Risk_Score = 13 if SBP[`i'] == "120-139" in `i'
            replace CVD_Risk_Score = 16 if SBP[`i'] == "140-159" in `i'
            replace CVD_Risk_Score = 20 if SBP[`i'] == "160-179" in `i'
            replace CVD_Risk_Score = 24 if SBP[`i'] == ">=180" in `i'
        }
         else if BMI[`i'] == ">=35" {
            replace CVD_Risk_Score = 12 if SBP[`i'] == "<120" in `i'
            replace CVD_Risk_Score = 14 if SBP[`i'] == "120-139" in `i'
            replace CVD_Risk_Score = 18 if SBP[`i'] == "140-159" in `i'
            replace CVD_Risk_Score = 22 if SBP[`i'] == "160-179" in `i'
            replace CVD_Risk_Score = 26 if SBP[`i'] == ">=180" in `i'
        }
    }
    else if Age_cat[`i'] == 4 {
        * Check the condition for BMI and update the CVD risk score based on systolic blood pressure
        if BMI[`i'] == "<20" {
            replace CVD_Risk_Score = 6 if SBP[`i'] == "<120" in `i'
            replace CVD_Risk_Score = 7 if SBP[`i'] == "120-139" in `i'
            replace CVD_Risk_Score = 9 if SBP[`i'] == "140-159" in `i'
            replace CVD_Risk_Score = 12 if SBP[`i'] == "160-179" in `i'
            replace CVD_Risk_Score = 15 if SBP[`i'] == ">=180" in `i'
        }
        else if BMI[`i'] == "20-24" {
            replace CVD_Risk_Score = 6 if SBP[`i'] == "<120" in `i'
            replace CVD_Risk_Score = 8 if SBP[`i'] == "120-139" in `i'
            replace CVD_Risk_Score = 10 if SBP[`i'] == "140-159" in `i'
            replace CVD_Risk_Score = 13 if SBP[`i'] == "160-179" in `i'
            replace CVD_Risk_Score = 16 if SBP[`i'] == ">=180" in `i'
        }
        else if BMI[`i'] == "25-29" {
            replace CVD_Risk_Score = 7 if SBP[`i'] == "<120" in `i'
            replace CVD_Risk_Score = 9 if SBP[`i'] == "120-139" in `i'
            replace CVD_Risk_Score = 11 if SBP[`i'] == "140-159" in `i'
            replace CVD_Risk_Score = 14 if SBP[`i'] == "160-179" in `i'
            replace CVD_Risk_Score = 18 if SBP[`i'] == ">=180" in `i'
        }
        else if BMI[`i'] == "30-35" {
            replace CVD_Risk_Score = 8 if SBP[`i'] == "<120" in `i'
            replace CVD_Risk_Score = 10 if SBP[`i'] == "120-139" in `i'
            replace CVD_Risk_Score = 13 if SBP[`i'] == "140-159" in `i'
            replace CVD_Risk_Score = 16 if SBP[`i'] == "160-179" in `i'
            replace CVD_Risk_Score = 20 if SBP[`i'] == ">=180" in `i'
        }
         else if BMI[`i'] == ">=35" {
            replace CVD_Risk_Score = 9 if SBP[`i'] == "<120" in `i'
            replace CVD_Risk_Score = 11 if SBP[`i'] == "120-139" in `i'
            replace CVD_Risk_Score = 14 if SBP[`i'] == "140-159" in `i'
            replace CVD_Risk_Score = 18 if SBP[`i'] == "160-179" in `i'
            replace CVD_Risk_Score = 22 if SBP[`i'] == ">=180" in `i'
```

```
        }
    }
    else if Age_cat[`i'] == 3 {
        * Check the condition for BMI and update the CVD risk score based on systolic blood pressure
        if BMI[`i'] == "<20" {
            replace CVD_Risk_Score = 4 if SBP[`i'] == "<120" in `i'
            replace CVD_Risk_Score = 5 if SBP[`i'] == "120-139" in `i'
            replace CVD_Risk_Score = 7 if SBP[`i'] == "140-159" in `i'
            replace CVD_Risk_Score = 9 if SBP[`i'] == "160-179" in `i'
            replace CVD_Risk_Score = 11 if SBP[`i'] == ">=180" in `i'
        }
        else if BMI[`i'] == "20-24" {
            replace CVD_Risk_Score = 5 if SBP[`i'] == "<120" in `i'
            replace CVD_Risk_Score = 6 if SBP[`i'] == "120-139" in `i'
            replace CVD_Risk_Score = 8 if SBP[`i'] == "140-159" in `i'
            replace CVD_Risk_Score = 10 if SBP[`i'] == "160-179" in `i'
            replace CVD_Risk_Score = 13 if SBP[`i'] == ">=180" in `i'
        }
        else if BMI[`i'] == "25-29" {
            replace CVD_Risk_Score = 5 if SBP[`i'] == "<120" in `i'
            replace CVD_Risk_Score = 7 if SBP[`i'] == "120-139" in `i'
            replace CVD_Risk_Score = 9 if SBP[`i'] == "140-159" in `i'
            replace CVD_Risk_Score = 11 if SBP[`i'] == "160-179" in `i'
            replace CVD_Risk_Score = 14 if SBP[`i'] == ">=180" in `i'
        }
        else if BMI[`i'] == "30-35" {
            replace CVD_Risk_Score = 6 if SBP[`i'] == "<120" in `i'
            replace CVD_Risk_Score = 8 if SBP[`i'] == "120-139" in `i'
            replace CVD_Risk_Score = 10 if SBP[`i'] == "140-159" in `i'
            replace CVD_Risk_Score = 13 if SBP[`i'] == "160-179" in `i'
            replace CVD_Risk_Score = 16 if SBP[`i'] == ">=180" in `i'
        }
         else if BMI[`i'] == ">=35" {
            replace CVD_Risk_Score = 7 if SBP[`i'] == "<120" in `i'
            replace CVD_Risk_Score = 9 if SBP[`i'] == "120-139" in `i'
            replace CVD_Risk_Score = 11 if SBP[`i'] == "140-159" in `i'
            replace CVD_Risk_Score = 14 if SBP[`i'] == "160-179" in `i'
            replace CVD_Risk_Score = 18 if SBP[`i'] == ">=180" in `i'
        }
    }
    else if Age_cat[`i'] == 2 {
        * Check the condition for BMI and update the CVD risk score based on systolic blood pressure
        if BMI[`i'] == "<20" {
            replace CVD_Risk_Score = 3 if SBP[`i'] == "<120" in `i'
            replace CVD_Risk_Score = 4 if SBP[`i'] == "120-139" in `i'
            replace CVD_Risk_Score = 5 if SBP[`i'] == "140-159" in `i'
            replace CVD_Risk_Score = 7 if SBP[`i'] == "160-179" in `i'
            replace CVD_Risk_Score = 9 if SBP[`i'] == ">=180" in `i'
        }
        else if BMI[`i'] == "20-24" {
            replace CVD_Risk_Score = 3 if SBP[`i'] == "<120" in `i'
            replace CVD_Risk_Score = 4 if SBP[`i'] == "120-139" in `i'
            replace CVD_Risk_Score = 6 if SBP[`i'] == "140-159" in `i'
            replace CVD_Risk_Score = 8 if SBP[`i'] == "160-179" in `i'
            replace CVD_Risk_Score = 10 if SBP[`i'] == ">=180" in `i'
        }
        else if BMI[`i'] == "25-29" {
            replace CVD_Risk_Score = 4 if SBP[`i'] == "<120" in `i'
            replace CVD_Risk_Score = 5 if SBP[`i'] == "120-139" in `i'
            replace CVD_Risk_Score = 7 if SBP[`i'] == "140-159" in `i'
            replace CVD_Risk_Score = 9 if SBP[`i'] == "160-179" in `i'
            replace CVD_Risk_Score = 11 if SBP[`i'] == ">=180" in `i'
        }
        else if BMI[`i'] == "30-35" {
            replace CVD_Risk_Score = 4 if SBP[`i'] == "<120" in `i'
            replace CVD_Risk_Score = 6 if SBP[`i'] == "120-139" in `i'
```

```
            replace CVD_Risk_Score = 8 if SBP[`i'] == "140-159" in `i'
            replace CVD_Risk_Score = 10 if SBP[`i'] == "160-179" in `i'
            replace CVD_Risk_Score = 13 if SBP[`i'] == ">=180" in `i'
        }
         else if BMI[`i'] == ">=35" {
            replace CVD_Risk_Score = 5 if SBP[`i'] == "<120" in `i'
            replace CVD_Risk_Score = 7 if SBP[`i'] == "120-139" in `i'
            replace CVD_Risk_Score = 9 if SBP[`i'] == "140-159" in `i'
            replace CVD_Risk_Score = 11 if SBP[`i'] == "160-179" in `i'
            replace CVD_Risk_Score = 15 if SBP[`i'] == ">=180" in `i'
        }
    }
    else if Age_cat[`i'] == 1 {
        * Check the condition for BMI and update the CVD risk score based on systolic blood pressure
        if BMI[`i'] == "<20" {
            replace CVD_Risk_Score = 2 if SBP[`i'] == "<120" in `i'
            replace CVD_Risk_Score = 3 if SBP[`i'] == "120-139" in `i'
            replace CVD_Risk_Score = 4 if SBP[`i'] == "140-159" in `i'
            replace CVD_Risk_Score = 5 if SBP[`i'] == "160-179" in `i'
            replace CVD_Risk_Score = 7 if SBP[`i'] == ">=180" in `i'
        }
        else if BMI[`i'] == "20-24" {
            replace CVD_Risk_Score = 2 if SBP[`i'] == "<120" in `i'
            replace CVD_Risk_Score = 3 if SBP[`i'] == "120-139" in `i'
            replace CVD_Risk_Score = 4 if SBP[`i'] == "140-159" in `i'
            replace CVD_Risk_Score = 6 if SBP[`i'] == "160-179" in `i'
            replace CVD_Risk_Score = 8 if SBP[`i'] == ">=180" in `i'
        }
        else if BMI[`i'] == "25-29" {
            replace CVD_Risk_Score = 3 if SBP[`i'] == "<120" in `i'
            replace CVD_Risk_Score = 4 if SBP[`i'] == "120-139" in `i'
            replace CVD_Risk_Score = 5 if SBP[`i'] == "140-159" in `i'
            replace CVD_Risk_Score = 7 if SBP[`i'] == "160-179" in `i'
            replace CVD_Risk_Score = 9 if SBP[`i'] == ">=180" in `i'
        }
        else if BMI[`i'] == "30-35" {
            replace CVD_Risk_Score = 3 if SBP[`i'] == "<120" in `i'
            replace CVD_Risk_Score = 4 if SBP[`i'] == "120-139" in `i'
            replace CVD_Risk_Score = 6 if SBP[`i'] == "140-159" in `i'
            replace CVD_Risk_Score = 8 if SBP[`i'] == "160-179" in `i'
            replace CVD_Risk_Score = 11 if SBP[`i'] == ">=180" in `i'
        }
         else if BMI[`i'] == ">=35" {
            replace CVD_Risk_Score = 4 if SBP[`i'] == "<120" in `i'
            replace CVD_Risk_Score = 5 if SBP[`i'] == "120-139" in `i'
            replace CVD_Risk_Score = 7 if SBP[`i'] == "140-159" in `i'
            replace CVD_Risk_Score = 9 if SBP[`i'] == "160-179" in `i'
            replace CVD_Risk_Score = 12 if SBP[`i'] == ">=180" in `i'
        }
    }
    else if Age_cat[`i'] == "40-44" {
        * Check the condition for BMI and update the CVD risk score based on systolic blood pressure
        if BMI[`i'] == "<20" {
            replace CVD_Risk_Score = 2 if SBP[`i'] == "<120" in `i'
            replace CVD_Risk_Score = 2 if SBP[`i'] == "120-139" in `i'
            replace CVD_Risk_Score = 3 if SBP[`i'] == "140-159" in `i'
            replace CVD_Risk_Score = 4 if SBP[`i'] == "160-179" in `i'
            replace CVD_Risk_Score = 5 if SBP[`i'] == ">=180" in `i'
        }
        else if BMI[`i'] == "20-24" {
            replace CVD_Risk_Score = 2 if SBP[`i'] == "<120" in `i'
            replace CVD_Risk_Score = 2 if SBP[`i'] == "120-139" in `i'
            replace CVD_Risk_Score = 3 if SBP[`i'] == "140-159" in `i'
            replace CVD_Risk_Score = 5 if SBP[`i'] == "160-179" in `i'
            replace CVD_Risk_Score = 6 if SBP[`i'] == ">=180" in `i'
        }
```

```
        else if BMI[`i'] == "25-29" {
            replace CVD_Risk_Score = 2 if SBP[`i'] == "<120" in `i'
            replace CVD_Risk_Score = 3 if SBP[`i'] == "120-139" in `i'
            replace CVD_Risk_Score = 4 if SBP[`i'] == "140-159" in `i'
            replace CVD_Risk_Score = 5 if SBP[`i'] == "160-179" in `i'
            replace CVD_Risk_Score = 7 if SBP[`i'] == ">=180" in `i'
        }
        else if BMI[`i'] == "30-35" {
            replace CVD_Risk_Score = 2 if SBP[`i'] == "<120" in `i'
            replace CVD_Risk_Score = 3 if SBP[`i'] == "120-139" in `i'
            replace CVD_Risk_Score = 5 if SBP[`i'] == "140-159" in `i'
            replace CVD_Risk_Score = 6 if SBP[`i'] == "160-179" in `i'
            replace CVD_Risk_Score = 9 if SBP[`i'] == ">=180" in `i'
        }
         else if BMI[`i'] == ">=35" {
            replace CVD_Risk_Score = 3 if SBP[`i'] == "<120" in `i'
            replace CVD_Risk_Score = 4 if SBP[`i'] == "120-139" in `i'
            replace CVD_Risk_Score = 5 if SBP[`i'] == "140-159" in `i'
            replace CVD_Risk_Score = 7 if SBP[`i'] == "160-179" in `i'
            replace CVD_Risk_Score = 10 if SBP[`i'] == ">=180" in `i'
        }
    }
   }
else if Smoking_status[`i'] == 1 {
       if Age_cat[`i'] == 6 {
        * Check the condition for BMI and update the CVD risk score based on systolic blood pressure
        if BMI[`i'] == "<20" {
            replace CVD_Risk_Score = 15 if SBP[`i'] == "<120" in `i'
            replace CVD_Risk_Score = 18 if SBP[`i'] == "120-139" in `i'
            replace CVD_Risk_Score = 22 if SBP[`i'] == "140-159" in `i'
            replace CVD_Risk_Score = 26 if SBP[`i'] == "160-179" in `i'
            replace CVD_Risk_Score = 31 if SBP[`i'] == ">=180" in `i'
        }
        else if BMI[`i'] == "20-24" {
            replace CVD_Risk_Score = 16 if SBP[`i'] == "<120" in `i'
            replace CVD_Risk_Score = 19 if SBP[`i'] == "120-139" in `i'
            replace CVD_Risk_Score = 23 if SBP[`i'] == "140-159" in `i'
            replace CVD_Risk_Score = 28 if SBP[`i'] == "160-179" in `i'
            replace CVD_Risk_Score = 33 if SBP[`i'] == ">=180" in `i'
        }
        else if BMI[`i'] == "25-29" {
            replace CVD_Risk_Score = 17 if SBP[`i'] == "<120" in `i'
            replace CVD_Risk_Score = 21 if SBP[`i'] == "120-139" in `i'
            replace CVD_Risk_Score = 25 if SBP[`i'] == "140-159" in `i'
            replace CVD_Risk_Score = 30 if SBP[`i'] == "160-179" in `i'
            replace CVD_Risk_Score = 35 if SBP[`i'] == ">=180" in `i'
        }
        else if BMI[`i'] == "30-35" {
            replace CVD_Risk_Score = 18 if SBP[`i'] == "<120" in `i'
            replace CVD_Risk_Score = 22 if SBP[`i'] == "120-139" in `i'
            replace CVD_Risk_Score = 27 if SBP[`i'] == "140-159" in `i'
            replace CVD_Risk_Score = 32 if SBP[`i'] == "160-179" in `i'
            replace CVD_Risk_Score = 37 if SBP[`i'] == ">=180" in `i'
        }
         else if BMI[`i'] == ">=35" {
            replace CVD_Risk_Score = 20 if SBP[`i'] == "<120" in `i'
            replace CVD_Risk_Score = 24 if SBP[`i'] == "120-139" in `i'
            replace CVD_Risk_Score = 28 if SBP[`i'] == "140-159" in `i'
            replace CVD_Risk_Score = 34 if SBP[`i'] == "160-179" in `i'
            replace CVD_Risk_Score = 40 if SBP[`i'] == ">=180" in `i'
        }
    }
    else if Age_cat[`i'] == 5 {
        * Check the condition for BMI and update the CVD risk score based on systolic blood pressure
        if BMI[`i'] == "<20" {
            replace CVD_Risk_Score = 11 if SBP[`i'] == "<120" in `i'
```

```
            replace CVD_Risk_Score = 14 if SBP[`i'] == "120-139" in `i'
            replace CVD_Risk_Score = 17 if SBP[`i'] == "140-159" in `i'
            replace CVD_Risk_Score = 21 if SBP[`i'] == "160-179" in `i'
            replace CVD_Risk_Score = 26 if SBP[`i'] == ">=180" in `i'
        }
        else if BMI[`i'] == "20-24" {
            replace CVD_Risk_Score = 12 if SBP[`i'] == "<120" in `i'
            replace CVD_Risk_Score = 15 if SBP[`i'] == "120-139" in `i'
            replace CVD_Risk_Score = 19 if SBP[`i'] == "140-159" in `i'
            replace CVD_Risk_Score = 23 if SBP[`i'] == "160-179" in `i'
            replace CVD_Risk_Score = 28 if SBP[`i'] == ">=180" in `i'
        }
        else if BMI[`i'] == "25-29" {
            replace CVD_Risk_Score = 14 if SBP[`i'] == "<120" in `i'
            replace CVD_Risk_Score = 17 if SBP[`i'] == "120-139" in `i'
            replace CVD_Risk_Score = 21 if SBP[`i'] == "140-159" in `i'
            replace CVD_Risk_Score = 25 if SBP[`i'] == "160-179" in `i'
            replace CVD_Risk_Score = 30 if SBP[`i'] == ">=180" in `i'
        }
        else if BMI[`i'] == "30-35" {
            replace CVD_Risk_Score = 15 if SBP[`i'] == "<120" in `i'
            replace CVD_Risk_Score = 18 if SBP[`i'] == "120-139" in `i'
            replace CVD_Risk_Score = 22 if SBP[`i'] == "140-159" in `i'
            replace CVD_Risk_Score = 27 if SBP[`i'] == "160-179" in `i'
            replace CVD_Risk_Score = 33 if SBP[`i'] == ">=180" in `i'
        }
         else if BMI[`i'] == ">=35" {
            replace CVD_Risk_Score = 16 if SBP[`i'] == "<120" in `i'
            replace CVD_Risk_Score = 20 if SBP[`i'] == "120-139" in `i'
            replace CVD_Risk_Score = 25 if SBP[`i'] == "140-159" in `i'
            replace CVD_Risk_Score = 30 if SBP[`i'] == "160-179" in `i'
            replace CVD_Risk_Score = 36 if SBP[`i'] == ">=180" in `i'
        }
    }
    else if Age_cat[`i'] == 4 {
        * Check the condition for BMI and update the CVD risk score based on systolic blood pressure
        if BMI[`i'] == "<20" {
            replace CVD_Risk_Score = 9 if SBP[`i'] == "<120" in `i'
            replace CVD_Risk_Score = 11 if SBP[`i'] == "120-139" in `i'
            replace CVD_Risk_Score = 14 if SBP[`i'] == "140-159" in `i'
            replace CVD_Risk_Score = 17 if SBP[`i'] == "160-179" in `i'
            replace CVD_Risk_Score = 21 if SBP[`i'] == ">=180" in `i'
        }
        else if BMI[`i'] == "20-24" {
            replace CVD_Risk_Score = 10 if SBP[`i'] == "<120" in `i'
            replace CVD_Risk_Score = 12 if SBP[`i'] == "120-139" in `i'
            replace CVD_Risk_Score = 15 if SBP[`i'] == "140-159" in `i'
            replace CVD_Risk_Score = 19 if SBP[`i'] == "160-179" in `i'
            replace CVD_Risk_Score = 24 if SBP[`i'] == ">=180" in `i'
        }
        else if BMI[`i'] == "25-29" {
            replace CVD_Risk_Score = 11 if SBP[`i'] == "<120" in `i'
            replace CVD_Risk_Score = 14 if SBP[`i'] == "120-139" in `i'
            replace CVD_Risk_Score = 17 if SBP[`i'] == "140-159" in `i'
            replace CVD_Risk_Score = 21 if SBP[`i'] == "160-179" in `i'
            replace CVD_Risk_Score = 26 if SBP[`i'] == ">=180" in `i'
        }
        else if BMI[`i'] == "30-35" {
            replace CVD_Risk_Score = 12 if SBP[`i'] == "<120" in `i'
            replace CVD_Risk_Score = 15 if SBP[`i'] == "120-139" in `i'
            replace CVD_Risk_Score = 19 if SBP[`i'] == "140-159" in `i'
            replace CVD_Risk_Score = 24 if SBP[`i'] == "160-179" in `i'
            replace CVD_Risk_Score = 29 if SBP[`i'] == ">=180" in `i'
        }
         else if BMI[`i'] == ">=35" {
            replace CVD_Risk_Score = 13 if SBP[`i'] == "<120" in `i'
            replace CVD_Risk_Score = 17 if SBP[`i'] == "120-139" in `i'
```

```
                replace CVD_Risk_Score = 21 if SBP[`i'] == "140-159" in `i'
                replace CVD_Risk_Score = 26 if SBP[`i'] == "160-179" in `i'
                replace CVD_Risk_Score = 32 if SBP[`i'] == ">=180" in `i'
            }
        }
        else if Age_cat[`i'] == 3 {
            * Check the condition for BMI and update the CVD risk score based on systolic blood pressure
            if BMI[`i'] == "<20" {
                replace CVD_Risk_Score = 7 if SBP[`i'] == "<120" in `i'
                replace CVD_Risk_Score = 9 if SBP[`i'] == "120-139" in `i'
                replace CVD_Risk_Score = 11 if SBP[`i'] == "140-159" in `i'
                replace CVD_Risk_Score = 14 if SBP[`i'] == "160-179" in `i'
                replace CVD_Risk_Score = 18 if SBP[`i'] == ">=180" in `i'
            }
            else if BMI[`i'] == "20-24" {
               replace CVD_Risk_Score = 7 if SBP[`i'] == "<120" in `i'
               replace CVD_Risk_Score = 10 if SBP[`i'] == "120-139" in `i'
               replace CVD_Risk_Score = 12 if SBP[`i'] == "140-159" in `i'
               replace CVD_Risk_Score = 16 if SBP[`i'] == "160-179" in `i'
               replace CVD_Risk_Score = 20 if SBP[`i'] == ">=180" in `i'
            }
            else if BMI[`i'] == "25-29" {
                replace CVD_Risk_Score = 8 if SBP[`i'] == "<120" in `i'
                replace CVD_Risk_Score = 11 if SBP[`i'] == "120-139" in `i'
                replace CVD_Risk_Score = 14 if SBP[`i'] == "140-159" in `i'
                replace CVD_Risk_Score = 28 if SBP[`i'] == "160-179" in `i'
                replace CVD_Risk_Score = 23 if SBP[`i'] == ">=180" in `i'
            }
            else if BMI[`i'] == "30-35" {
                replace CVD_Risk_Score = 10 if SBP[`i'] == "<120" in `i'
                replace CVD_Risk_Score = 12 if SBP[`i'] == "120-139" in `i'
                replace CVD_Risk_Score = 16 if SBP[`i'] == "140-159" in `i'
                replace CVD_Risk_Score = 20 if SBP[`i'] == "160-179" in `i'
                replace CVD_Risk_Score = 26 if SBP[`i'] == ">=180" in `i'
            }
            else if BMI[`i'] == ">=35" {
                replace CVD_Risk_Score = 11 if SBP[`i'] == "<120" in `i'
                replace CVD_Risk_Score = 14 if SBP[`i'] == "120-139" in `i'
                replace CVD_Risk_Score = 18 if SBP[`i'] == "140-159" in `i'
                replace CVD_Risk_Score = 23 if SBP[`i'] == "160-179" in `i'
                replace CVD_Risk_Score = 29 if SBP[`i'] == ">=180" in `i'
            }
        }
        else if Age_cat[`i'] == 2 {
            * Check the condition for BMI and update the CVD risk score based on systolic blood pressure
            if BMI[`i'] == "<20" {
                replace CVD_Risk_Score = 5 if SBP[`i'] == "<120" in `i'
                replace CVD_Risk_Score = 7 if SBP[`i'] == "120-139" in `i'
                replace CVD_Risk_Score = 9 if SBP[`i'] == "140-159" in `i'
                replace CVD_Risk_Score = 11 if SBP[`i'] == "160-179" in `i'
                replace CVD_Risk_Score = 15 if SBP[`i'] == ">=180" in `i'
            }
            else if BMI[`i'] == "20-24" {
                replace CVD_Risk_Score = 6 if SBP[`i'] == "<120" in `i'
                replace CVD_Risk_Score = 8 if SBP[`i'] == "120-139" in `i'
                replace CVD_Risk_Score = 10 if SBP[`i'] == "140-159" in `i'
                replace CVD_Risk_Score = 13 if SBP[`i'] == "160-179" in `i'
                replace CVD_Risk_Score = 17 if SBP[`i'] == ">=180" in `i'
            }
            else if BMI[`i'] == "25-29" {
                replace CVD_Risk_Score = 7 if SBP[`i'] == "<120" in `i'
                replace CVD_Risk_Score = 9 if SBP[`i'] == "120-139" in `i'
                replace CVD_Risk_Score = 12 if SBP[`i'] == "140-159" in `i'
                replace CVD_Risk_Score = 15 if SBP[`i'] == "160-179" in `i'
                replace CVD_Risk_Score = 20 if SBP[`i'] == ">=180" in `i'
            }
```

```
        else if BMI[`i'] == "30-35" {
            replace CVD_Risk_Score = 8 if SBP[`i'] == "<120" in `i'
            replace CVD_Risk_Score = 10 if SBP[`i'] == "120-139" in `i'
            replace CVD_Risk_Score = 13 if SBP[`i'] == "140-159" in `i'
            replace CVD_Risk_Score = 17 if SBP[`i'] == "160-179" in `i'
            replace CVD_Risk_Score = 22 if SBP[`i'] == ">=180" in `i'
        }
         else if BMI[`i'] == ">=35" {
            replace CVD_Risk_Score = 9 if SBP[`i'] == "<120" in `i'
            replace CVD_Risk_Score = 12 if SBP[`i'] == "120-139" in `i'
            replace CVD_Risk_Score = 15 if SBP[`i'] == "140-159" in `i'
            replace CVD_Risk_Score = 20 if SBP[`i'] == "160-179" in `i'
            replace CVD_Risk_Score = 26 if SBP[`i'] == ">=180" in `i'
        }
    }
    else if Age_cat[`i'] == 1 {
        * Check the condition for BMI and update the CVD risk score based on systolic blood pressure
        if BMI[`i'] == "<20" {
            replace CVD_Risk_Score = 4 if SBP[`i'] == "<120" in `i'
            replace CVD_Risk_Score = 5 if SBP[`i'] == "120-139" in `i'
            replace CVD_Risk_Score = 7 if SBP[`i'] == "140-159" in `i'
            replace CVD_Risk_Score = 9 if SBP[`i'] == "160-179" in `i'
            replace CVD_Risk_Score = 12 if SBP[`i'] == ">=180" in `i'
        }
        else if BMI[`i'] == "20-24" {
            replace CVD_Risk_Score = 4 if SBP[`i'] == "<120" in `i'
            replace CVD_Risk_Score = 6 if SBP[`i'] == "120-139" in `i'
            replace CVD_Risk_Score = 8 if SBP[`i'] == "140-159" in `i'
            replace CVD_Risk_Score = 11 if SBP[`i'] == "160-179" in `i'
            replace CVD_Risk_Score = 14 if SBP[`i'] == ">=180" in `i'
        }
        else if BMI[`i'] == "25-29" {
            replace CVD_Risk_Score = 5 if SBP[`i'] == "<120" in `i'
            replace CVD_Risk_Score = 7 if SBP[`i'] == "120-139" in `i'
            replace CVD_Risk_Score = 9 if SBP[`i'] == "140-159" in `i'
            replace CVD_Risk_Score = 13 if SBP[`i'] == "160-179" in `i'
            replace CVD_Risk_Score = 17 if SBP[`i'] == ">=180" in `i'
        }
        else if BMI[`i'] == "30-35" {
            replace CVD_Risk_Score = 6 if SBP[`i'] == "<120" in `i'
            replace CVD_Risk_Score = 8 if SBP[`i'] == "120-139" in `i'
            replace CVD_Risk_Score = 11 if SBP[`i'] == "140-159" in `i'
            replace CVD_Risk_Score = 15 if SBP[`i'] == "160-179" in `i'
            replace CVD_Risk_Score = 20 if SBP[`i'] == ">=180" in `i'
        }
         else if BMI[`i'] == ">=35" {
            replace CVD_Risk_Score = 7 if SBP[`i'] == "<120" in `i'
            replace CVD_Risk_Score = 10 if SBP[`i'] == "120-139" in `i'
            replace CVD_Risk_Score = 13 if SBP[`i'] == "140-159" in `i'
            replace CVD_Risk_Score = 17 if SBP[`i'] == "160-179" in `i'
            replace CVD_Risk_Score = 23 if SBP[`i'] == ">=180" in `i'
        }
    }
    else if Age_cat[`i'] == "40-44" {
        * Check the condition for BMI and update the CVD risk score based on systolic blood pressure
        if BMI[`i'] == "<20" {
            replace CVD_Risk_Score = 3 if SBP[`i'] == "<120"  in `i'
            replace CVD_Risk_Score = 4 if SBP[`i'] == "120-139" in `i'
            replace CVD_Risk_Score = 5 if SBP[`i'] == "140-159" in `i'
            replace CVD_Risk_Score = 8 if SBP[`i'] == "160-179" in `i'
            replace CVD_Risk_Score = 10 if SBP[`i'] == ">=180" in `i'
        }
        else if BMI[`i'] == "20-24" {
            replace CVD_Risk_Score = 3 if SBP[`i'] == "<120" in `i'
            replace CVD_Risk_Score = 5 if SBP[`i'] == "120-139" in `i'
            replace CVD_Risk_Score = 6 if SBP[`i'] == "140-159" in `i'
```

```
            replace CVD_Risk_Score = 9 if SBP[`i'] == "160-179" in `i'
            replace CVD_Risk_Score = 12 if SBP[`i'] == ">=180" in `i'
        }
        else if BMI[`i'] == "25-29" {
            replace CVD_Risk_Score = 4 if SBP[`i'] == "<120" in `i'
            replace CVD_Risk_Score = 6 if SBP[`i'] == "120-139" in `i'
            replace CVD_Risk_Score = 8 if SBP[`i'] == "140-159" in `i'
            replace CVD_Risk_Score = 11 if SBP[`i'] == "160-179" in `i'
            replace CVD_Risk_Score = 14 if SBP[`i'] == ">=180" in `i'
        }
        else if BMI[`i'] == "30-35" {
            replace CVD_Risk_Score = 5 if SBP[`i'] == "<120" in `i'
            replace CVD_Risk_Score = 7 if SBP[`i'] == "120-139" in `i'
            replace CVD_Risk_Score = 9 if SBP[`i'] == "140-159" in `i'
            replace CVD_Risk_Score = 13 if SBP[`i'] == "160-179" in `i'
            replace CVD_Risk_Score = 17 if SBP[`i'] == ">=180" in `i'
        }
         else if BMI[`i'] == ">=35" {
            replace CVD_Risk_Score = 6 if SBP[`i'] == "<120" in `i'
            replace CVD_Risk_Score = 8 if SBP[`i'] == "120-139" in `i'
            replace CVD_Risk_Score = 11 if SBP[`i'] == "140-159" in `i'
            replace CVD_Risk_Score = 15 if SBP[`i'] == "160-179" in `i'
            replace CVD_Risk_Score = 20 if SBP[`i'] == ">=180" in `i'
        }
    }
   }
}
else if Sex[`i'] == 2 {
        if Smoking_status[`i'] == 2 {
            if Age_cat[`i'] == 6 {
        * Check the condition for BMI and update the CVD risk score based on systolic blood pressure
        if BMI[`i'] == "<20" {
            replace CVD_Risk_Score = 10 if SBP[`i'] == "<120" in `i'
            replace CVD_Risk_Score = 12 if SBP[`i'] == "120-139" in `i'
            replace CVD_Risk_Score = 15 if SBP[`i'] == "140-159" in `i'
            replace CVD_Risk_Score = 17 if SBP[`i'] == "160-179" in `i'
            replace CVD_Risk_Score = 21 if SBP[`i'] == ">=180" in `i'
        }
        else if BMI[`i'] == "20-24" {
            replace CVD_Risk_Score = 11 if SBP[`i'] == "<120" in `i'
            replace CVD_Risk_Score = 13 if SBP[`i'] == "120-139" in `i'
            replace CVD_Risk_Score = 15 if SBP[`i'] == "140-159" in `i'
            replace CVD_Risk_Score = 18 if SBP[`i'] == "160-179" in `i'
            replace CVD_Risk_Score = 21 if SBP[`i'] == ">=180" in `i'
        }
        else if BMI[`i'] == "25-29" {
            replace CVD_Risk_Score = 11 if SBP[`i'] == "<120" in `i'
            replace CVD_Risk_Score = 13 if SBP[`i'] == "120-139" in `i'
            replace CVD_Risk_Score = 16 if SBP[`i'] == "140-159" in `i'
            replace CVD_Risk_Score = 19 if SBP[`i'] == "160-179" in `i'
            replace CVD_Risk_Score = 22 if SBP[`i'] == ">=180" in `i'
        }
        else if BMI[`i'] == "30-35" {
            replace CVD_Risk_Score = 12 if SBP[`i'] == "<120" in `i'
            replace CVD_Risk_Score = 14 if SBP[`i'] == "120-139" in `i'
            replace CVD_Risk_Score = 16 if SBP[`i'] == "140-159" in `i'
            replace CVD_Risk_Score = 19 if SBP[`i'] == "160-179" in `i'
            replace CVD_Risk_Score = 23 if SBP[`i'] == ">=180" in `i'
        }
         else if BMI[`i'] == ">=35" {
            replace CVD_Risk_Score = 12 if SBP[`i'] == "<120" in `i'
            replace CVD_Risk_Score = 14 if SBP[`i'] == "120-139" in `i'
            replace CVD_Risk_Score = 17 if SBP[`i'] == "140-159" in `i'
            replace CVD_Risk_Score = 20 if SBP[`i'] == "160-179" in `i'
            replace CVD_Risk_Score = 24 if SBP[`i'] == ">=180" in `i'
        }
    }
```

```
    else if Age_cat[`i'] == 5 {
        * Check the condition for BMI and update the CVD risk score based on systolic blood pressure
        if BMI[`i'] == "<20" {
            replace CVD_Risk_Score = 7 if SBP[`i'] == "<120" in `i'
            replace CVD_Risk_Score = 9 if SBP[`i'] == "120-139" in `i'
            replace CVD_Risk_Score = 11 if SBP[`i'] == "140-159" in `i'
            replace CVD_Risk_Score = 13 if SBP[`i'] == "160-179" in `i'
            replace CVD_Risk_Score = 16 if SBP[`i'] == ">=180" in `i'
        }
        else if BMI[`i'] == "20-24" {
            replace CVD_Risk_Score = 8 if SBP[`i'] == "<120" in `i'
            replace CVD_Risk_Score = 9 if SBP[`i'] == "120-139" in `i'
            replace CVD_Risk_Score = 11 if SBP[`i'] == "140-159" in `i'
            replace CVD_Risk_Score = 14 if SBP[`i'] == "160-179" in `i'
            replace CVD_Risk_Score = 17 if SBP[`i'] == ">=180" in `i'
        }
        else if BMI[`i'] == "25-29" {
            replace CVD_Risk_Score = 8 if SBP[`i'] == "<120" in `i'
            replace CVD_Risk_Score = 10 if SBP[`i'] == "120-139" in `i'
            replace CVD_Risk_Score = 12 if SBP[`i'] == "140-159" in `i'
            replace CVD_Risk_Score = 14 if SBP[`i'] == "160-179" in `i'
            replace CVD_Risk_Score = 18 if SBP[`i'] == ">=180" in `i'
        }
        else if BMI[`i'] == "30-35" {
            replace CVD_Risk_Score = 8 if SBP[`i'] == "<120" in `i'
            replace CVD_Risk_Score = 10 if SBP[`i'] == "120-139" in `i'
            replace CVD_Risk_Score = 12 if SBP[`i'] == "140-159" in `i'
            replace CVD_Risk_Score = 15 if SBP[`i'] == "160-179" in `i'
            replace CVD_Risk_Score = 18 if SBP[`i'] == ">=180" in `i'
        }
         else if BMI[`i'] == ">=35" {
            replace CVD_Risk_Score = 9 if SBP[`i'] == "<120" in `i'
            replace CVD_Risk_Score = 11 if SBP[`i'] == "120-139" in `i'
            replace CVD_Risk_Score = 13 if SBP[`i'] == "140-159" in `i'
            replace CVD_Risk_Score = 16 if SBP[`i'] == "160-179" in `i'
            replace CVD_Risk_Score = 19 if SBP[`i'] == ">=180" in `i'
        }
    }
    else if Age_cat[`i'] == 4 {
        * Check the condition for BMI and update the CVD risk score based on systolic blood pressure
        if BMI[`i'] == "<20" {
            replace CVD_Risk_Score = 5 if SBP[`i'] == "<120" in `i'
            replace CVD_Risk_Score = 7 if SBP[`i'] == "120-139" in `i'
            replace CVD_Risk_Score = 8 if SBP[`i'] == "140-159" in `i'
            replace CVD_Risk_Score = 10 if SBP[`i'] == "160-179" in `i'
            replace CVD_Risk_Score = 13 if SBP[`i'] == ">=180" in `i'
        }
        else if BMI[`i'] == "20-24" {
            replace CVD_Risk_Score = 5 if SBP[`i'] == "<120" in `i'
            replace CVD_Risk_Score = 7 if SBP[`i'] == "120-139" in `i'
            replace CVD_Risk_Score = 9 if SBP[`i'] == "140-159" in `i'
            replace CVD_Risk_Score = 11 if SBP[`i'] == "160-179" in `i'
            replace CVD_Risk_Score = 13 if SBP[`i'] == ">=180" in `i'
        }
        else if BMI[`i'] == "25-29" {
            replace CVD_Risk_Score = 6 if SBP[`i'] == "<120" in `i'
            replace CVD_Risk_Score = 7 if SBP[`i'] == "120-139" in `i'
            replace CVD_Risk_Score = 9 if SBP[`i'] == "140-159" in `i'
            replace CVD_Risk_Score = 11 if SBP[`i'] == "160-179" in `i'
            replace CVD_Risk_Score = 14 if SBP[`i'] == ">=180" in `i'
        }
        else if BMI[`i'] == "30-35" {
            replace CVD_Risk_Score = 6 if SBP[`i'] == "<120" in `i'
            replace CVD_Risk_Score = 8 if SBP[`i'] == "120-139" in `i'
            replace CVD_Risk_Score = 9 if SBP[`i'] == "140-159" in `i'
            replace CVD_Risk_Score = 12 if SBP[`i'] == "160-179" in `i'
```

```
            replace CVD_Risk_Score = 14 if SBP[`i'] == ">=180" in `i'
        }
         else if BMI[`i'] == ">=35" {
            replace CVD_Risk_Score = 6 if SBP[`i'] == "<120" in `i'
            replace CVD_Risk_Score = 8 if SBP[`i'] == "120-139" in `i'
            replace CVD_Risk_Score = 10 if SBP[`i'] == "140-159" in `i'
            replace CVD_Risk_Score = 12 if SBP[`i'] == "160-179" in `i'
            replace CVD_Risk_Score = 15 if SBP[`i'] == ">=180" in `i'
        }
    }
    else if Age_cat[`i'] == 3 {
        * Check the condition for BMI and update the CVD risk score based on systolic blood pressure
        if BMI[`i'] == "<20" {
            replace CVD_Risk_Score = 4 if SBP[`i'] == "<120" in `i'
            replace CVD_Risk_Score = 5 if SBP[`i'] == "120-139" in `i'
            replace CVD_Risk_Score = 6 if SBP[`i'] == "140-159" in `i'
            replace CVD_Risk_Score = 8 if SBP[`i'] == "160-179" in `i'
            replace CVD_Risk_Score = 10 if SBP[`i'] == ">=180" in `i'
        }
        else if BMI[`i'] == "20-24" {
            replace CVD_Risk_Score = 4 if SBP[`i'] == "<120" in `i'
            replace CVD_Risk_Score = 5 if SBP[`i'] == "120-139" in `i'
            replace CVD_Risk_Score = 6 if SBP[`i'] == "140-159" in `i'
            replace CVD_Risk_Score = 8 if SBP[`i'] == "160-179" in `i'
            replace CVD_Risk_Score = 10 if SBP[`i'] == ">=180" in `i'
        }
        else if BMI[`i'] == "25-29" {
            replace CVD_Risk_Score = 4 if SBP[`i'] == "<120" in `i'
            replace CVD_Risk_Score = 5 if SBP[`i'] == "120-139" in `i'
            replace CVD_Risk_Score = 7 if SBP[`i'] == "140-159" in `i'
            replace CVD_Risk_Score = 9 if SBP[`i'] == "160-179" in `i'
            replace CVD_Risk_Score = 11 if SBP[`i'] == ">=180" in `i'
        }
        else if BMI[`i'] == "30-35" {
            replace CVD_Risk_Score = 4 if SBP[`i'] == "<120" in `i'
            replace CVD_Risk_Score = 6 if SBP[`i'] == "120-139" in `i'
            replace CVD_Risk_Score = 7 if SBP[`i'] == "140-159" in `i'
            replace CVD_Risk_Score = 9 if SBP[`i'] == "160-179" in `i'
            replace CVD_Risk_Score = 11 if SBP[`i'] == ">=180" in `i'
        }
         else if BMI[`i'] == ">=35" {
            replace CVD_Risk_Score = 5 if SBP[`i'] == "<120" in `i'
            replace CVD_Risk_Score = 6 if SBP[`i'] == "120-139" in `i'
            replace CVD_Risk_Score = 7 if SBP[`i'] == "140-159" in `i'
            replace CVD_Risk_Score = 10 if SBP[`i'] == "160-179" in `i'
            replace CVD_Risk_Score = 12 if SBP[`i'] == ">=180" in `i'
        }
    }
    else if Age_cat[`i'] == 2 {
        * Check the condition for BMI and update the CVD risk score based on systolic blood pressure
        if BMI[`i'] == "<20" {
            replace CVD_Risk_Score = 3 if SBP[`i'] == "<120" in `i'
            replace CVD_Risk_Score = 3 if SBP[`i'] == "120-139" in `i'
            replace CVD_Risk_Score = 5 if SBP[`i'] == "140-159" in `i'
            replace CVD_Risk_Score = 6 if SBP[`i'] == "160-179" in `i'
            replace CVD_Risk_Score = 8 if SBP[`i'] == ">=180" in `i'
        }
        else if BMI[`i'] == "20-24" {
            replace CVD_Risk_Score = 3 if SBP[`i'] == "<120" in `i'
            replace CVD_Risk_Score = 4 if SBP[`i'] == "120-139" in `i'
            replace CVD_Risk_Score = 5 if SBP[`i'] == "140-159" in `i'
            replace CVD_Risk_Score = 6 if SBP[`i'] == "160-179" in `i'
            replace CVD_Risk_Score = 8 if SBP[`i'] == ">=180" in `i'
        }
        else if BMI[`i'] == "25-29" {
            replace CVD_Risk_Score = 3 if SBP[`i'] == "<120" in `i'
```

```
            replace CVD_Risk_Score = 4 if SBP[`i'] == "120-139" in `i'
            replace CVD_Risk_Score = 5 if SBP[`i'] == "140-159" in `i'
            replace CVD_Risk_Score = 7 if SBP[`i'] == "160-179" in `i'
            replace CVD_Risk_Score = 9 if SBP[`i'] == ">=180" in `i'
        }
        else if BMI[`i'] == "30-35" {
            replace CVD_Risk_Score = 3 if SBP[`i'] == "<120" in `i'
            replace CVD_Risk_Score = 4 if SBP[`i'] == "120-139" in `i'
            replace CVD_Risk_Score = 5 if SBP[`i'] == "140-159" in `i'
            replace CVD_Risk_Score = 7 if SBP[`i'] == "160-179" in `i'
            replace CVD_Risk_Score = 9 if SBP[`i'] == ">=180" in `i'
        }
         else if BMI[`i'] == ">=35" {
            replace CVD_Risk_Score = 3 if SBP[`i'] == "<120" in `i'
            replace CVD_Risk_Score = 4 if SBP[`i'] == "120-139" in `i'
            replace CVD_Risk_Score = 6 if SBP[`i'] == "140-159" in `i'
            replace CVD_Risk_Score = 7 if SBP[`i'] == "160-179" in `i'
            replace CVD_Risk_Score = 10 if SBP[`i'] == ">=180" in `i'
        }
    }
    else if Age_cat[`i'] == 1 {
        * Check the condition for BMI and update the CVD risk score based on systolic blood pressure
        if BMI[`i'] == "<20" {
            replace CVD_Risk_Score = 2 if SBP[`i'] == "<120" in `i'
            replace CVD_Risk_Score = 2 if SBP[`i'] == "120-139" in `i'
            replace CVD_Risk_Score = 3 if SBP[`i'] == "140-159" in `i'
            replace CVD_Risk_Score = 5 if SBP[`i'] == "160-179" in `i'
            replace CVD_Risk_Score = 6 if SBP[`i'] == ">=180" in `i'
        }
        else if BMI[`i'] == "20-24" {
            replace CVD_Risk_Score = 2 if SBP[`i'] == "<120" in `i'
            replace CVD_Risk_Score = 3 if SBP[`i'] == "120-139" in `i'
            replace CVD_Risk_Score = 4 if SBP[`i'] == "140-159" in `i'
            replace CVD_Risk_Score = 5 if SBP[`i'] == "160-179" in `i'
            replace CVD_Risk_Score = 6 if SBP[`i'] == ">=180" in `i'
        }
        else if BMI[`i'] == "25-29" {
            replace CVD_Risk_Score = 2 if SBP[`i'] == "<120" in `i'
            replace CVD_Risk_Score = 3 if SBP[`i'] == "120-139" in `i'
            replace CVD_Risk_Score = 4 if SBP[`i'] == "140-159" in `i'
            replace CVD_Risk_Score = 5 if SBP[`i'] == "160-179" in `i'
            replace CVD_Risk_Score = 7 if SBP[`i'] == ">=180" in `i'
        }
        else if BMI[`i'] == "30-35" {
            replace CVD_Risk_Score = 2 if SBP[`i'] == "<120" in `i'
            replace CVD_Risk_Score = 3 if SBP[`i'] == "120-139" in `i'
            replace CVD_Risk_Score = 4 if SBP[`i'] == "140-159" in `i'
            replace CVD_Risk_Score = 5 if SBP[`i'] == "160-179" in `i'
            replace CVD_Risk_Score = 7 if SBP[`i'] == ">=180" in `i'
        }
         else if BMI[`i'] == ">=35" {
            replace CVD_Risk_Score = 2 if SBP[`i'] == "<120" in `i'
            replace CVD_Risk_Score = 3 if SBP[`i'] == "120-139" in `i'
            replace CVD_Risk_Score = 4 if SBP[`i'] == "140-159" in `i'
            replace CVD_Risk_Score = 6 if SBP[`i'] == "160-179" in `i'
            replace CVD_Risk_Score = 8 if SBP[`i'] == ">=180" in `i'
        }
    }
    else if Age_cat[`i'] == "40-44" {
        * Check the condition for BMI and update the CVD risk score based on systolic blood pressure
        if BMI[`i'] == "<20" {
            replace CVD_Risk_Score = 1 if SBP[`i'] == "<120" in `i'
            replace CVD_Risk_Score = 2 if SBP[`i'] == "120-139" in `i'
            replace CVD_Risk_Score = 2 if SBP[`i'] == "140-159" in `i'
            replace CVD_Risk_Score = 3 if SBP[`i'] == "160-179" in `i'
            replace CVD_Risk_Score = 5 if SBP[`i'] == ">=180" in `i'
```

```
        }
        else if BMI[`i'] == "20-24" {
            replace CVD_Risk_Score = 1 if SBP[`i'] == "<120" in `i'
            replace CVD_Risk_Score = 2 if SBP[`i'] == "120-139" in `i'
            replace CVD_Risk_Score = 3 if SBP[`i'] == "140-159" in `i'
            replace CVD_Risk_Score = 4 if SBP[`i'] == "160-179" in `i'
            replace CVD_Risk_Score = 5 if SBP[`i'] == ">=180" in `i'
        }
        else if BMI[`i'] == "25-29" {
            replace CVD_Risk_Score = 1 if SBP[`i'] == "<120" in `i'
            replace CVD_Risk_Score = 2 if SBP[`i'] == "120-139" in `i'
            replace CVD_Risk_Score = 3 if SBP[`i'] == "140-159" in `i'
            replace CVD_Risk_Score = 4 if SBP[`i'] == "160-179" in `i'
            replace CVD_Risk_Score = 5 if SBP[`i'] == ">=180" in `i'
        }
        else if BMI[`i'] == "30-35" {
            replace CVD_Risk_Score = 1 if SBP[`i'] == "<120" in `i'
            replace CVD_Risk_Score = 2 if SBP[`i'] == "120-139" in `i'
            replace CVD_Risk_Score = 3 if SBP[`i'] == "140-159" in `i'
            replace CVD_Risk_Score = 4 if SBP[`i'] == "160-179" in `i'
            replace CVD_Risk_Score = 6 if SBP[`i'] == ">=180" in `i'
        }
        else if BMI[`i'] == ">=35" {
            replace CVD_Risk_Score = 2 if SBP[`i'] == "<120" in `i'
            replace CVD_Risk_Score = 2 if SBP[`i'] == "120-139" in `i'
            replace CVD_Risk_Score = 3 if SBP[`i'] == "140-159" in `i'
            replace CVD_Risk_Score = 4 if SBP[`i'] == "160-179" in `i'
            replace CVD_Risk_Score = 6 if SBP[`i'] == ">=180" in `i'
        }
    }
   }
else if Smoking_status[`i'] == 1 {
       if Age_cat[`i'] == 6 {
        * Check the condition for BMI and update the CVD risk score based on systolic blood pressure
        if BMI[`i'] == "<20" {
            replace CVD_Risk_Score = 15 if SBP[`i'] == "<120" in `i'
            replace CVD_Risk_Score = 18 if SBP[`i'] == "120-139" in `i'
            replace CVD_Risk_Score = 21 if SBP[`i'] == "140-159" in `i'
            replace CVD_Risk_Score = 25 if SBP[`i'] == "160-179" in `i'
            replace CVD_Risk_Score = 29 if SBP[`i'] == ">=180" in `i'
        }
        else if BMI[`i'] == "20-24" {
            replace CVD_Risk_Score = 15 if SBP[`i'] == "<120" in `i'
            replace CVD_Risk_Score = 18 if SBP[`i'] == "120-139" in `i'
            replace CVD_Risk_Score = 22 if SBP[`i'] == "140-159" in `i'
            replace CVD_Risk_Score = 26 if SBP[`i'] == "160-179" in `i'
            replace CVD_Risk_Score = 30 if SBP[`i'] == ">=180" in `i'
        }
        else if BMI[`i'] == "25-29" {
            replace CVD_Risk_Score = 16 if SBP[`i'] == "<120" in `i'
            replace CVD_Risk_Score = 19 if SBP[`i'] == "120-139" in `i'
            replace CVD_Risk_Score = 22 if SBP[`i'] == "140-159" in `i'
            replace CVD_Risk_Score = 26 if SBP[`i'] == "160-179" in `i'
            replace CVD_Risk_Score = 31 if SBP[`i'] == ">=180" in `i'
        }
        else if BMI[`i'] == "30-35" {
            replace CVD_Risk_Score = 17 if SBP[`i'] == "<120" in `i'
            replace CVD_Risk_Score = 20 if SBP[`i'] == "120-139" in `i'
            replace CVD_Risk_Score = 23 if SBP[`i'] == "140-159" in `i'
            replace CVD_Risk_Score = 27 if SBP[`i'] == "160-179" in `i'
            replace CVD_Risk_Score = 32 if SBP[`i'] == ">=180" in `i'
        }
         else if BMI[`i'] == ">=35" {
            replace CVD_Risk_Score = 17 if SBP[`i'] == "<120" in `i'
            replace CVD_Risk_Score = 20 if SBP[`i'] == "120-139" in `i'
            replace CVD_Risk_Score = 24 if SBP[`i'] == "140-159" in `i'
            replace CVD_Risk_Score = 28 if SBP[`i'] == "160-179" in `i'
```

```
            replace CVD_Risk_Score = 33 if SBP[`i'] == ">=180" in `i'
        }
    }
    else if Age_cat[`i'] == 5 {
        * Check the condition for BMI and update the CVD risk score based on systolic blood pressure
        if BMI[`i'] == "<20" {
            replace CVD_Risk_Score = 12 if SBP[`i'] == "<120" in `i'
            replace CVD_Risk_Score = 14 if SBP[`i'] == "120-139" in `i'
            replace CVD_Risk_Score = 17 if SBP[`i'] == "140-159" in `i'
            replace CVD_Risk_Score = 21 if SBP[`i'] == "160-179" in `i'
            replace CVD_Risk_Score = 25 if SBP[`i'] == ">=180" in `i'
        }
        else if BMI[`i'] == "20-24" {
            replace CVD_Risk_Score = 12 if SBP[`i'] == "<120" in `i'
            replace CVD_Risk_Score = 15 if SBP[`i'] == "120-139" in `i'
            replace CVD_Risk_Score = 18 if SBP[`i'] == "140-159" in `i'
            replace CVD_Risk_Score = 21 if SBP[`i'] == "160-179" in `i'
            replace CVD_Risk_Score = 26 if SBP[`i'] == ">=180" in `i'
        }
        else if BMI[`i'] == "25-29" {
            replace CVD_Risk_Score = 13 if SBP[`i'] == "<120" in `i'
            replace CVD_Risk_Score = 15 if SBP[`i'] == "120-139" in `i'
            replace CVD_Risk_Score = 19 if SBP[`i'] == "140-159" in `i'
            replace CVD_Risk_Score = 22 if SBP[`i'] == "160-179" in `i'
            replace CVD_Risk_Score = 27 if SBP[`i'] == ">=180" in `i'
        }
        else if BMI[`i'] == "30-35" {
            replace CVD_Risk_Score = 13 if SBP[`i'] == "<120" in `i'
            replace CVD_Risk_Score = 16 if SBP[`i'] == "120-139" in `i'
            replace CVD_Risk_Score = 19 if SBP[`i'] == "140-159" in `i'
            replace CVD_Risk_Score = 23 if SBP[`i'] == "160-179" in `i'
            replace CVD_Risk_Score = 28 if SBP[`i'] == ">=180" in `i'
        }
         else if BMI[`i'] == ">=35" {
            replace CVD_Risk_Score = 14 if SBP[`i'] == "<120" in `i'
            replace CVD_Risk_Score = 17 if SBP[`i'] == "120-139" in `i'
            replace CVD_Risk_Score = 20 if SBP[`i'] == "140-159" in `i'
            replace CVD_Risk_Score = 24 if SBP[`i'] == "160-179" in `i'
            replace CVD_Risk_Score = 29 if SBP[`i'] == ">=180" in `i'
        }
    }
    else if Age_cat[`i'] == 4 {
        * Check the condition for BMI and update the CVD risk score based on systolic blood pressure
        if BMI[`i'] == "<20" {
            replace CVD_Risk_Score = 9 if SBP[`i'] == "<120" in `i'
            replace CVD_Risk_Score = 11 if SBP[`i'] == "120-139" in `i'
            replace CVD_Risk_Score = 14 if SBP[`i'] == "140-159" in `i'
            replace CVD_Risk_Score = 17 if SBP[`i'] == "160-179" in `i'
            replace CVD_Risk_Score = 21 if SBP[`i'] == ">=180" in `i'
        }
        else if BMI[`i'] == "20-24" {
            replace CVD_Risk_Score = 9 if SBP[`i'] == "<120" in `i'
            replace CVD_Risk_Score = 12 if SBP[`i'] == "120-139" in `i'
            replace CVD_Risk_Score = 15 if SBP[`i'] == "140-159" in `i'
            replace CVD_Risk_Score = 18 if SBP[`i'] == "160-179" in `i'
            replace CVD_Risk_Score = 22 if SBP[`i'] == ">=180" in `i'
        }
        else if BMI[`i'] == "25-29" {
            replace CVD_Risk_Score = 10 if SBP[`i'] == "<120" in `i'
            replace CVD_Risk_Score = 12 if SBP[`i'] == "120-139" in `i'
            replace CVD_Risk_Score = 15 if SBP[`i'] == "140-159" in `i'
            replace CVD_Risk_Score = 19 if SBP[`i'] == "160-179" in `i'
            replace CVD_Risk_Score = 23 if SBP[`i'] == ">=180" in `i'
        }
        else if BMI[`i'] == "30-35" {
            replace CVD_Risk_Score = 11 if SBP[`i'] == "<120" in `i'
```

```
                replace CVD_Risk_Score = 13 if SBP[`i'] == "120-139" in `i'
                replace CVD_Risk_Score = 16 if SBP[`i'] == "140-159" in `i'
                replace CVD_Risk_Score = 20 if SBP[`i'] == "160-179" in `i'
                replace CVD_Risk_Score = 24 if SBP[`i'] == ">=180" in `i'
            }
             else if BMI[`i'] == ">=35" {
                replace CVD_Risk_Score = 11 if SBP[`i'] == "<120" in `i'
                replace CVD_Risk_Score = 14 if SBP[`i'] == "120-139" in `i'
                replace CVD_Risk_Score = 17 if SBP[`i'] == "140-159" in `i'
                replace CVD_Risk_Score = 21 if SBP[`i'] == "160-179" in `i'
                replace CVD_Risk_Score = 26 if SBP[`i'] == ">=180" in `i'
            }
        }
        else if Age_cat[`i'] == 3 {
            * Check the condition for BMI and update the CVD risk score based on systolic blood pressure
            if BMI[`i'] == "<20" {
                replace CVD_Risk_Score = 7 if SBP[`i'] == "<120" in `i'
                replace CVD_Risk_Score = 9 if SBP[`i'] == "120-139" in `i'
                replace CVD_Risk_Score = 11 if SBP[`i'] == "140-159" in `i'
                replace CVD_Risk_Score = 14 if SBP[`i'] == "160-179" in `i'
                replace CVD_Risk_Score = 18 if SBP[`i'] == ">=180" in `i'
            }
            else if BMI[`i'] == "20-24" {
                replace CVD_Risk_Score = 7 if SBP[`i'] == "<120" in `i'
                replace CVD_Risk_Score = 9 if SBP[`i'] == "120-139" in `i'
                replace CVD_Risk_Score = 12 if SBP[`i'] == "140-159" in `i'
                replace CVD_Risk_Score = 15 if SBP[`i'] == "160-179" in `i'
                replace CVD_Risk_Score = 19 if SBP[`i'] == ">=180" in `i'
            }
            else if BMI[`i'] == "25-29" {
                replace CVD_Risk_Score = 8 if SBP[`i'] == "<120" in `i'
                replace CVD_Risk_Score = 10 if SBP[`i'] == "120-139" in `i'
                replace CVD_Risk_Score = 13 if SBP[`i'] == "140-159" in `i'
                replace CVD_Risk_Score = 16 if SBP[`i'] == "160-179" in `i'
                replace CVD_Risk_Score = 20 if SBP[`i'] == ">=180" in `i'
            }
            else if BMI[`i'] == "30-35" {
                replace CVD_Risk_Score = 8 if SBP[`i'] == "<120" in `i'
                replace CVD_Risk_Score = 11 if SBP[`i'] == "120-139" in `i'
                replace CVD_Risk_Score = 13 if SBP[`i'] == "140-159" in `i'
                replace CVD_Risk_Score = 17 if SBP[`i'] == "160-179" in `i'
                replace CVD_Risk_Score = 21 if SBP[`i'] == ">=180" in `i'
            }
             else if BMI[`i'] == ">=35" {
                replace CVD_Risk_Score = 9 if SBP[`i'] == "<120" in `i'
                replace CVD_Risk_Score = 11 if SBP[`i'] == "120-139" in `i'
                replace CVD_Risk_Score = 14 if SBP[`i'] == "140-159" in `i'
                replace CVD_Risk_Score = 18 if SBP[`i'] == "160-179" in `i'
                replace CVD_Risk_Score = 22 if SBP[`i'] == ">=180" in `i'
            }
        }
        else if Age_cat[`i'] == 2 {
            * Check the condition for BMI and update the CVD risk score based on systolic blood pressure
            if BMI[`i'] == "<20" {
                replace CVD_Risk_Score = 5 if SBP[`i'] == "<120" in `i'
                replace CVD_Risk_Score = 7 if SBP[`i'] == "120-139" in `i'
                replace CVD_Risk_Score = 9 if SBP[`i'] == "140-159" in `i'
                replace CVD_Risk_Score = 12 if SBP[`i'] == "160-179" in `i'
                replace CVD_Risk_Score = 15 if SBP[`i'] == ">=180" in `i'
            }
            else if BMI[`i'] == "20-24" {
                replace CVD_Risk_Score = 6 if SBP[`i'] == "<120" in `i'
                replace CVD_Risk_Score = 7 if SBP[`i'] == "120-139" in `i'
                replace CVD_Risk_Score = 10 if SBP[`i'] == "140-159" in `i'
                replace CVD_Risk_Score = 13 if SBP[`i'] == "160-179" in `i'
                replace CVD_Risk_Score = 16 if SBP[`i'] == ">=180" in `i'
```

```
        }
        else if BMI[`i'] == "25-29" {
            replace CVD_Risk_Score = 6 if SBP[`i'] == "<120" in `i'
            replace CVD_Risk_Score = 8 if SBP[`i'] == "120-139" in `i'
            replace CVD_Risk_Score = 10 if SBP[`i'] == "140-159" in `i'
            replace CVD_Risk_Score = 13 if SBP[`i'] == "160-179" in `i'
            replace CVD_Risk_Score = 17 if SBP[`i'] == ">=180" in `i'
        }
        else if BMI[`i'] == "30-35" {
            replace CVD_Risk_Score = 7 if SBP[`i'] == "<120" in `i'
            replace CVD_Risk_Score = 9 if SBP[`i'] == "120-139" in `i'
            replace CVD_Risk_Score = 11 if SBP[`i'] == "140-159" in `i'
            replace CVD_Risk_Score = 14 if SBP[`i'] == "160-179" in `i'
            replace CVD_Risk_Score = 18 if SBP[`i'] == ">=180" in `i'
        }
         else if BMI[`i'] == ">=35" {
            replace CVD_Risk_Score = 7 if SBP[`i'] == "<120" in `i'
            replace CVD_Risk_Score = 9 if SBP[`i'] == "120-139" in `i'
            replace CVD_Risk_Score = 12 if SBP[`i'] == "140-159" in `i'
            replace CVD_Risk_Score = 15 if SBP[`i'] == "160-179" in `i'
            replace CVD_Risk_Score = 19 if SBP[`i'] == ">=180" in `i'
        }
    }
    else if Age_cat[`i'] == 1 {
        * Check the condition for BMI and update the CVD risk score based on systolic blood pressure
        if BMI[`i'] == "<20" {
            replace CVD_Risk_Score = 4 if SBP[`i'] == "<120" in `i'
            replace CVD_Risk_Score = 6 if SBP[`i'] == "120-139" in `i'
            replace CVD_Risk_Score = 7 if SBP[`i'] == "140-159" in `i'
            replace CVD_Risk_Score = 10 if SBP[`i'] == "160-179" in `i'
            replace CVD_Risk_Score = 13 if SBP[`i'] == ">=180" in `i'
        }
        else if BMI[`i'] == "20-24" {
            replace CVD_Risk_Score = 4 if SBP[`i'] == "<120" in `i'
            replace CVD_Risk_Score = 6 if SBP[`i'] == "120-139" in `i'
            replace CVD_Risk_Score = 8 if SBP[`i'] == "140-159" in `i'
            replace CVD_Risk_Score = 10 if SBP[`i'] == "160-179" in `i'
            replace CVD_Risk_Score = 14 if SBP[`i'] == ">=180" in `i'
        }
        else if BMI[`i'] == "25-29" {
            replace CVD_Risk_Score = 5 if SBP[`i'] == "<120" in `i'
            replace CVD_Risk_Score = 6 if SBP[`i'] == "120-139" in `i'
            replace CVD_Risk_Score = 8 if SBP[`i'] == "140-159" in `i'
            replace CVD_Risk_Score = 11 if SBP[`i'] == "160-179" in `i'
            replace CVD_Risk_Score = 15 if SBP[`i'] == ">=180" in `i'
        }
        else if BMI[`i'] == "30-35" {
            replace CVD_Risk_Score = 5 if SBP[`i'] == "<120" in `i'
            replace CVD_Risk_Score = 7 if SBP[`i'] == "120-139" in `i'
            replace CVD_Risk_Score = 9 if SBP[`i'] == "140-159" in `i'
            replace CVD_Risk_Score = 12 if SBP[`i'] == "160-179" in `i'
            replace CVD_Risk_Score = 16 if SBP[`i'] == ">=180" in `i'
        }
         else if BMI[`i'] == ">=35" {
            replace CVD_Risk_Score = 6 if SBP[`i'] == "<120" in `i'
            replace CVD_Risk_Score = 7 if SBP[`i'] == "120-139" in `i'
            replace CVD_Risk_Score = 10 if SBP[`i'] == "140-159" in `i'
            replace CVD_Risk_Score = 13 if SBP[`i'] == "160-179" in `i'
            replace CVD_Risk_Score = 17 if SBP[`i'] == ">=180" in `i'
        }
    }
    else if Age_cat[`i'] == "40-44" {
        * Check the condition for BMI and update the CVD risk score based on systolic blood pressure
        if BMI[`i'] == "<20" {
            replace CVD_Risk_Score = 3 if SBP[`i'] == "<120" in `i'
            replace CVD_Risk_Score = 4 if SBP[`i'] == "120-139" in `i'
```

```
            replace CVD_Risk_Score = 6 if SBP[`i'] == "140-159" in `i'
            replace CVD_Risk_Score = 8 if SBP[`i'] == "160-179" in `i'
            replace CVD_Risk_Score = 11 if SBP[`i'] == ">=180" in `i'
        }
        else if BMI[`i'] == "20-24" {
            replace CVD_Risk_Score = 3 if SBP[`i'] == "<120" in `i'
            replace CVD_Risk_Score = 5 if SBP[`i'] == "120-139" in `i'
            replace CVD_Risk_Score = 6 if SBP[`i'] == "140-159" in `i'
            replace CVD_Risk_Score = 9 if SBP[`i'] == "160-179" in `i'
            replace CVD_Risk_Score = 12 if SBP[`i'] == ">=180" in `i'
        }
        else if BMI[`i'] == "25-29" {
            replace CVD_Risk_Score = 4 if SBP[`i'] == "<120" in `i'
            replace CVD_Risk_Score = 5 if SBP[`i'] == "120-139" in `i'
            replace CVD_Risk_Score = 7 if SBP[`i'] == "140-159" in `i'
            replace CVD_Risk_Score = 9 if SBP[`i'] == "160-179" in `i'
            replace CVD_Risk_Score = 13 if SBP[`i'] == ">=180" in `i'
        }
        else if BMI[`i'] == "30-35" {
            replace CVD_Risk_Score = 4 if SBP[`i'] == "<120" in `i'
            replace CVD_Risk_Score = 6 if SBP[`i'] == "120-139" in `i'
            replace CVD_Risk_Score = 7 if SBP[`i'] == "140-159" in `i'
            replace CVD_Risk_Score = 10 if SBP[`i'] == "160-179" in `i'
            replace CVD_Risk_Score = 14 if SBP[`i'] == ">=180" in `i'
        }
         else if BMI[`i'] == ">=35" {
            replace CVD_Risk_Score = 4 if SBP[`i'] == "<120" in `i'
            replace CVD_Risk_Score = 6 if SBP[`i'] == "120-139" in `i'
            replace CVD_Risk_Score = 8 if SBP[`i'] == "140-159" in `i'
            replace CVD_Risk_Score = 11 if SBP[`i'] == "160-179" in `i'
            replace CVD_Risk_Score = 15 if SBP[`i'] == ">=180" in `i'
        }
    }
   }
}
}
```
